# Cultural specificity and linguistic diversity in medical nutrition therapy education resources for type 2 diabetes

**DOI:** 10.3389/fcdhc.2026.1823506

**Published:** 2026-05-15

**Authors:** Olivia Y. Wu, Anna Schwerdfeger, Tamara R. Cohen

**Affiliations:** 1 Food, Nutrition, and Health, Faculty of Land and Food Systems, University of British Columbia, Vancouver, BC, Canada; 2 British Columbia Children’s Hospital Research Institute, Healthy Starts, British Columbia (BC) Children’s Hospital, Vancouver, BC, Canada

**Keywords:** culturally competent care, dietetics, medical nutrition therapy, patient education, type 2 diabetes

## Abstract

**Introduction:**

Culturally tailored nutrition care improves diabetes-related health outcomes, yet little is known about the cultural specificity and multilingual accessibility of education resources used in medical nutrition therapy for type 2 diabetes. This mini review examined the cultural specificity and linguistic diversity of Canadian diabetes nutrition therapy resources and compared resources’ characteristics based on the presence of these features.

**Methods:**

A mini review adopting systematic search methods for gray literature was conducted. Data sources include bibliographic and gray literature databases, customized search engines, targeted sites, and consultations with dietitian diabetes educators. Eligible resources were freely available online, in English, and published by Canadian organizations. Resources’ cultural specificity, language availability, year, publisher, format, and contents were extracted. Quality was assessed using Authority, Accuracy, Coverage, Objectivity, Date, Significance checklist.

**Results:**

We identified 341 (46 professional- and 295 patient-facing) resources. A third of resources (37% of professional and 28% of patient) contained culturally specific guidance, most frequently for Indigenous and South and Southeast Asian groups. Guidance for African, Caribbean, Middle Eastern, East Asian, and Latin American groups was sparse. Just 9% of patient resources were available in languages other than English or French, and 4% had both cultural specificity and third-language translations. Culturally specific professional resources had less comprehensive content coverage than general resources. Culturally or linguistically diverse patient resources were comprehensive but often outdated.

**Discussion:**

Professional and patient education resources with expanded cultural diversity and multilingual options are needed to support medical nutrition therapy for ethnoculturally diverse Canadians with type 2 diabetes.

## 1 Introduction

Medical nutrition therapy (MNT) describes evidence-based nutrition interventions delivered by registered dietitians (RDs), and is essential to type 2 diabetes (T2D) management (1). Canadian dietitians serve a highly diverse population, including ethnocultural groups with disproportionately higher rates of T2D compared to White populations (2, 3). Among South Asian, Black, and First Nations adults, diabetes prevalence is 2.3, 2.1, and 1.0 times higher than among White adults, respectively (3). Culturally tailored and language concordant diabetes care improves clinical outcomes and dietary behaviors among ethnocultural groups (4–10), underscoring the importance of clinicians equipped to provide culturally responsive MNT.

Professional and patient education resources are important tools that support MNT delivery. Clinicians rely on clinical practice guidelines and other professional resources that synthesize evidence to inform practice (11–13). Patient education materials provide accessible information that can enhance patients’ understanding of clinical guidance, as well as their health literacy and self-efficacy (14–16). However, little is known about the cultural specificity and multilingual availability of education resources used in Canadian MNT for T2D.

Understanding the cultural specificity and linguistic diversity of MNT for T2D resources can provide a benchmark of resources available to clinicians and inform gaps between current offerings and needs. Identifying any discrepancies in the characteristics and contents of culturally specific or linguistically diverse and general resources can support future resource development for equitable and relevant nutrition diabetes care. This study aimed to identify professional and patient education resources relevant to Canadian MNT for T2D, describe resources’ cultural specificity and language availability, and compare resources’ descriptive characteristics and content based on their inclusion of cultural specificity and multilingual options.

## 2 Materials and methods

Professional resources were defined as materials intended to inform Canadian RDs’ provision of MNT for adults living with T2D. Patient resources were defined as materials to support dietary practices that improve diabetes-related health outcomes in Canadians living with T2D. Resources were included if they contained text and/or images, were transcripts of audiovisual material, were publicly available online, free, in English, and published by Canadian organizations Resources primarily intended for people living with type 1 diabetes, pregnant individuals, children, or dependent adults, those that focused on nutrients without mention of foods, and those replaced by newer versions of the same resource were excluded.

This mini review adopted Godin et al.’s approach to applying systematic search strategies to gray literature, which utilizes four search strategies: gray literature databases, customized Google search engines, targeted websites, and consultation with knowledge experts (17). An additional strategy, bibliographic databases, was included to identify any relevant peer-reviewed articles. For bibliographic databases, MEDLINE (Ovid), CINAHL, and PubMed were used. For gray literature databases, CPG Infobase, Guidelines International Network, ClinicalKey, Turning Research into Practice (TRIP) Database, and BC Guidelines and Protocols were used. The Canadian Public Health Information customized Google search engine was used to narrow Google search results to Canadian federal and provincial health departments and other public health agencies (18). Targeted websites of relevant organizations and agencies were identified based on the first and last author’s clinical experiences and hand-searched for relevant resources.

Registered dietitian diabetes educators (RD CDEs) providing MNT to independent adults living with T2D were consulted as knowledge experts to identify resources used in practice. We purposefully sampled RD CDEs across Canada to include representation from various provinces, territories, and population center sizes using professional networks and the Canadian Diabetes Educator Certification Board website. RD CDEs participated in a 15-to-30-minute semi-structured phone or video conferencing call where they were asked to share the professional and patient resources commonly used in their practices, or shared resources via email. Emails were sent to 138 RD CDEs across Canada, of which 26 were consulted. RD CDEs practiced in small (19%; n=5), medium (15%, n=4), and large population centers (65%; n=17) across all provinces and Nunavut; no RD CDEs were available from the Northwestern Territories and Yukon.

The review process was documented through a study flow diagram conforming to Preferred Reporting Items for Systematic Reviews and Meta-Analyses (17, 19). Screening and full text review were conducted by one reviewer between March and July 2023. Data extraction and quality appraisal were conducted by two reviewers between June and August 2023. Resources deemed relevant after reviewing their titles and source organizations were imported into Zotero 6.0.36 (Corporation for Digital Scholarship, VA, USA) and Covidence (Veritas Health Innovation, Melbourne, Australia). The abstracts, summaries, and/or table of contents of each resource were then screened for full-text review. Duplicates were excluded through Covidence and manually. Documents for which eligibility was unclear were conservatively included for full-text review. When it was unclear whether a resource met the eligibility criteria during full-text review, the senior author was consulted to reach consensus.

Two reviewers independently extracted resources’ cultural specificity, language availability, year of publication, type of publishing organization, format, and inclusion of nutritional management of diabetes-related comorbidities and MNT topics. Resources were considered culturally specific if they described food choices, meal planning strategies, or nutrition counselling approaches tailored to named cultural groups; resources that broadly discussed the need to consider cultural acceptability in MNT were not classified as such. Specific cultural groups targeted by resources were extracted verbatim. Language availability referred to whether resources were offered in languages other than English, and, if so, which languages were provided. Categories used for extraction of nutritional management of diabetes-related comorbidities and MNT topics were determined *a priori* based on the 2018 Diabetes Canada Clinical Practice Guidelines, a professional-facing MNT for T2D resource, and the first and last authors’ clinical experiences (20). Categories that were not initially included but relevant were incorporated iteratively during data extraction. Two reviewers independently assessed each resource using the Authority, Accuracy, Coverage, Objectivity, Date, Significance (ACCODS) checklist (21) to exclude resources of questionable quality that would likely not be used in clinical practice (22). Any discrepancies in data extraction and quality appraisal were resolved by discussion. Additional information on all search strategies, data extraction, and quality appraisal is available in **Supplementary Materials**

All data extracted were categorical variables and summarized using frequencies and proportions. Comparisons of proportions of descriptive characteristics and inclusion of cultural specificity and linguistic diversity among professional and patient resources was conducted using Fisher’s Exact test in Stata SE 18.5 (StataCorp, TX, USA). Null hypotheses were set as the absence of differences across comparison groups, with a significance of p<0.05.

Review by the institutional review board was not required for this study because human subjects were not involved, as per the Tri-Council Policy Statement: Ethical Conduct for Research Involving Humans (TCPS-2) (23).

## 3 Results

A total of 341 (46 professional- and 295 patient-facing) resources met the inclusion criteria. Two RD CDEs serving primarily Indigenous populations noted that printed resources were rarely used and that interactive workshops and sharing circles were preferred for nutrition education. A study flow diagram and complete list of resources are available in **Supplementary Materials**.

### 3.1 Descriptive characteristics

Most professional and patient resources were published between 2018 and 2023 (**Table 1**). Professional resources were primarily published by national NGOs, followed by provincial or territorial governmental organizations. Patient resources were most often published by national NGOs and provincial or territorial NGOs. Professional resources were mostly in the form of clinical practice guidelines, while patient resources were mostly handouts.

**Table 1:** Characteristics of professional and patient medical nutrition therapy resources for type 2 diabetes.

Characteristics	Total		Professional		Patient		P-value
	n	%	n	%	n	%	
n (%) of all resources	341	(100)	46	(13)	295	(87)	
Cultural specificity							
No	240	(70)	29	(63)	211	(72)	
Yes	101	(30)	17	(37)	84	(28)	
African	8	(2)	1	(2)	7	(2)	
Caribbean	9	(3)	0	(0)	9	(3)	
East Asian	10	(3)	2	(4)	8	(3)	
Indigenous	51	(15)	9	(20)	42	(14)	
Latin American	6	(2)	1	(2)	5	(2)	
Middle Eastern	3	(1)	2	(4)	1	(0)	0.049
Muslim (Ramadan)	6	(2)	6	(13)	0	(0)	<0.001
South & Southeast Asian	22	(6)	3	(7)	19	(6)	
Translations available							
No	213	(63)	34	(74)	179	(61)	
French	116	(34)	12	(26)	104	(35)	
Other (Third languages)	28	(6)	0	(0)	28	(9)	0.021
Both cultural specificity and third language availability							
No	313	(92)	46	(13)	284	(91)	<0.001
Yes	28	(8)	0	(0)	11	(9)	<0.001
Year published							
2018 to 2023	226	(68)	39	(85)	191	(65)	0.007
2012 to 2017	79	(23)	5	(11)	74	(25)	0.038
2007 to 2011	13	(4)	1	(2)	12	(4)	
Unknown	23	(7)	1	(2)	12	(4)	
Type of publishing organization							
National NGO	169	(50)	36	(78)	133	(45)	<0.001
National government	3	(1)	0	(0)	3	(1)	
Provincial or territorial NGO	104	(30)	1	(2)	103	(35)	<0.001
Provincial or territorial government	65	(19)	9	(20)	56	(19)	
Type of resource							
Clinical practice guideline	24	(7)	24	(52)	0	(0)	<0.001
Position statement	2	(1)	2	(4)	0	(0)	0.018
Report	11	(3)	5	(11)	6	(2)	0.009
Handout	209	(61)	7	(15)	202	(68)	<0.001
Web article	71	(2)	0	(0)	71	(24)	<0.001
Audiovisual	24	(7)	8	(17)	16	(5)	0.008
Nutrition management of diabetes-related comorbidities							
Blood sugar management	341	(100)	46	(100)	295	(100)	
Weight management	124	(36)	35	(76)	89	(30)	<0.001
Cardiovascular disease	144	(42)	33	(72)	111	(38)	<0.001
Hypertension	76	(22)	25	(54)	51	(17)	<0.001
Gastrointestinal health	39	(11)	1	(2)	38	(13)	0.042
Gastroparesis	7	(2)	1	(2)	6	(2)	
Nephropathy	43	(13)	16	(35)	27	(9)	<0.001
Neuropathy	18	(5)	3	(7)	15	(5)	
Retinopathy	18	(5)	3	(7)	15	(5)	
Mental health	64	(19)	26	(57)	38	(13)	<0.001
Medical nutrition therapy topics							
General healthy eating	68	(20)	7	(15)	61	(21)	
Meal planning	172	(50)	16	(35)	156	(53)	0.026
Snack planning	142	(42)	9	(20)	133	(45)	<0.001
Macronutrients	298	(87)	36	(78)	262	(89)	0.045
Micronutrients	135	(40)	23	(50)	112	(38)	
Carbohydrate counting	62	(18)	5	(11)	57	(19)	
Label reading	128	(38)	8	(17)	120	(41)	0.003
Eating away from home	49	(14)	2	(4)	47	(16)	0.040
Non-nutritive sweeteners	52	(15)	8	(17)	44	(15)	
Dietary patterns	176	(52)	29	(63)	147	(50)	
Mediterranean diet	27	(8)	14	(30)	13	(4)	<0.001
Glycemic index	62	(18)	16	(35)	46	(16)	0.003
Low carbohydrate diet	19	(6)	17	(37)	2	(1)	<0.001
Canada's Food Guide	59	(17)	10	(22)	49	(17)	
Intermittent fasting	8	(2)	7	(15)	1	(0)	<0.001
Low calorie diet	8	(2)	8	(17)	0	(0)	<0.001
DASH diet	23	(7)	10	(22)	13	(4)	<0.001
Low fat diet	10	(3)	10	(22)	0	(0)	<0.001
Nordic diet	4	(1)	2	(4)	2	(1)	0.089
Popular weight loss diets	7	(2)	5	(11)	2	(1)	<0.001
Balanced Plate Method	84	(25)	5	(11)	79	(27)	0.017
Gluten-free diet	6	(2)	0	(0)	6	(2)	
Vegetarian diet	31	(9)	8	(17)	23	(8)	
Portfolio diet	8	(2)	5	(11)	3	(1)	<0.001

NGO non-governmental organization. Data expressed as number (percent of total resources per column). Comparisons were made between percentages of professional and patient resources in each category using Fisher’s exact test. Percentages in “cultural specificity”, “translations available”, “nutrition management of diabetes-related comorbidities” and “medical nutrition therapy topics” do not total 100% because individual resources may fall into multiple categories. P-values not noted were ≥0.05.

A larger proportion of professional resources included nutrition management for diabetes-related comorbidities compared to patient resources. Nutritional contexts to managing cardiovascular disease, weight, hypertension, and mental health were more prevalent in professional resources than in patient resources. Nephropathy appeared in over one-third of professional resources but only 9% of patient resources (p<0.01). Gastroparesis, neuropathy, and retinopathy were least frequently addressed across both resource types (< 7%).

Macronutrients was the most common MNT topic in both professional and patient resources. Professional resources also frequently included dietary patterns and micronutrients, while patient resources typically emphasized meal planning, snack planning, and label reading. Patient resources were more likely to address practical topics such as eating away from home. Professional resources were more likely to reference certain dietary patterns, including low carbohydrate, low glycemic index, and Mediterranean diets. The Balanced Plate method was more commonly found in patient resources.

### 3.2 Cultural specificity and multilingual availability

Roughly one-third of resources included culturally specific nutrition guidance (37% professional versus 28% patient) (**Table 1**). The cultural groups most frequently represented were Indigenous (20% versus 14%) and South or Southeast Asian populations (7% versus 6%). Guidance for African, Caribbean, Middle Eastern, East Asian, and Latin American groups was low (<5%) across professional and patient resources. Guidance for Muslim populations appeared in 13% of professional resources but was not found in patient resources (p<0.01). Most culturally specific patient resources targeted broad cultural groups. Among South and Southeast Asian resources, six resources (32%) distinguished between North Indian (n=3) and South Indian (n=3) or Gujarati (n=1) groups, while thirteen (68%) targeted the broader cultural group. No patient resources for African, Caribbean or Middle Eastern populations identified guidance for cultural subgroups. The exception was for East Asian resources, where seven out of eight resources (88%) offered guidance specific to Chinese populations.

Most resources were available only in English, with French translations available in 26% of professional and 35% patient resources. No professional resources were available in other languages. Nine percent of patient resources offered third-language translations, comprising 17 languages, including Mandarin (Traditional n=14; Simplified n=9), Urdu (n=10), Punjabi (n=9), Hindi (n=8), Spanish (n=7), Tamil (n=7), Arabic (n=5), Ojibwe (n=5), Plains Cree (n=5), Portuguese (n=5), Inuktitut (n=4), Oji-Cree (n=3), Polish (n=3), Inuinnaqtun (n=2), Italian (n=2), Tagalog (n=2), Farsi-Persian (n=1). Just 4% (n=11) of patient resources combined cultural specificity with third-language translations. Resources with both cultural specificity and third-language translations targeted South and Southeast Asian (n=4), East Asian (n=3), Indigenous (n=3), and Latin American (n=1) groups.

### 3.3 Comparing culturally specific, multilingual, and other resources

Culturally specific professional resources were more likely than general resources to be published between 2012 and 2017, although this difference was not statistically significant (p=0.055). Culturally specific professional resources were also less likely to include discussions of weight management and cardiovascular disease compared to general resources. For MNT topics, professional resources with cultural specificity were more likely to include meal planning, but less likely to include the Mediterranean, low carbohydrate, and low-calorie diets.

Culturally specific patient resources were more likely than non-culturally specific resources to be published between 2007 and 2017 and in the form of handouts, but less likely to be presented as web articles. Inclusion of MNT topics and diabetes management goals was largely similar across culturally tailored and non-tailored patient resources. Whenever differences appeared, a larger proportion of culturally tailored resources included the topic. Topics with differing inclusion include mental health, meal planning, snack planning, and dietary patterns, such as Canada’s Food Guide, the Balanced Plate Method, and vegetarian diets.

Patient resources with and without third-language translations did not have significant differences in publication dates. However, patient resources with third-language translations were more likely to be published by national NGOs than by provincial or territorial NGOs or government agencies. Compared to general resources, culturally specific patient resources were more often presented as handouts and less often as web articles. Inclusion of MNT and disease management topics were similar across resources with or without third-language translations, and translation-available resources had greater inclusion of topics when differences emerged. Topics with differing inclusion between resources with or without third-language translations include carbohydrate counting and dietary patterns, such as popular weight loss diets and the Balanced Plate Method.

Compared to patient resources with neither cultural specificity nor third-language translations, a larger proportion of resources with both features were published before 2011 and in the form of handouts. Resources with both features were more likely to mention carbohydrate counting and dietary patterns, but less likely to mention label reading.

Detailed comparisons of resource characteristics by cultural specificity and language availability are available in **Supplementary Materials**.

## 4 Discussion

This mini review described the cultural specificity and multilingual availability of 341 professional and patient education resources available for Canadian MNT for T2D. While roughly a third of resources contained culturally relevant guidance, only 9% of patient resources were available in languages other than English or French, and just 4% combined both features. Culturally specific professional resources had less comprehensive coverage of comorbidity management goals and MNT topics compared to general professional resources. Culturally tailored patient resources and those with both cultural specificity and third-language translations were comprehensive in content, but older.

The volume of culturally specific resources identified is encouraging, but gaps remain for underrepresented groups. With over 450 reported ethnic or cultural origins, one-quarter of Canadians are visible minorities and one-quarter are immigrants, primarily from Asia and Africa (24, 25). While Indigenous and South and Southeast Asian groups were most frequently represented in culturally specific diabetes nutrition resources, guidance for populations with high risk of diabetes-related morbidity and mortality, such as African groups, was sparse (2, 3, 26, 27). In addition, although MNT can mitigate the potential for adverse hypo- and hyperglycemic episodes during Ramadan (28, 29), no resources were available to support patient-facing nutrition education for Muslim populations. This finding aligns with Hillier et al.’s survey of 157 Canadian RDs, where 69% of Canadian dietitians serving Muslim clients during Ramadan reported lacking appropriate resources (30).

The variety of third-language translations available in patient resources aligns with Canada’s most common non-official languages (Mandarin, Urdu, Punjabi, Hindi, Tamil) (31). However, the limited number of resources with third-language translations raises concerns about whether they are accessible or sufficient. Over 450 languages are spoken in Canada (24), and close to half speak a language other than English or French most often at home (31). Language barriers hinder T2D self-management (32, 33) whereas language-concordant care improves cardiovascular outcomes (34) and acceptance of lifestyle interventions (35). Further, most third-language resources lacked cultural tailoring, with only 39% providing culturally specific guidance. Patients who require or prefer resources with third-language translations are more likely to be immigrants, a group that is more likely to face higher risks of diabetes, and tends to place greater importance on their ethnic and cultural origins than non-immigrants (24, 36). These patients would be better served by resources that integrate cultural tailoring with multilingual options rather than verbatim translations of general nutrition resources.

Culturally specific professional resources had less comprehensive coverage of nutritional management of diabetes-related comorbidities and MNT topics than non-culturally specific professional resources. The extent to which this may affect culturally sensitive diabetes care depends on the combination of MNT for T2D resources that clinicians use and on their broader cultural competency. As culturally specific professional resources may supplement other professional MNT guidelines, their less comprehensive coverage of comorbidity-related nutrition management may not necessarily indicate gaps in the information clinicians provide to ethnically diverse patients. However, the lack of culturally tailored guidance for MNT topics may be clinically relevant. Patient resources often described the importance of adapting dietary patterns to cultural contexts but provided little concrete guidance, implicitly assuming that RDs who access these resources possess the necessary skills to make these adaptations. Cultural competency is a core component of dietetic education (22), but research among Canadian RDs highlights gaps in training. Over 90% of RDs who responded to a survey about culturally safe care for Muslims reported not receiving relevant education during their university degree (30). Undergraduate dietetic students reported similar shortcomings in cultural competency training (37). Among RDs working in T2D care, a considerable 32% disagreed that the 2018 Diabetes Canada’s Clinical Practice Guidelines can be easily adapted to meet ethnocultural client needs (13). Improved cultural specificity in professional-facing diabetes nutrition resources and expanded provision of cultural skills in dietetic education are likely needed to support clinicians working with diverse populations living with T2D.

In contrast to professional resources, patient resources with cultural specificity and third-language translations were generally comprehensive in comorbidity management and MNT topics, with higher likelihoods of discussing dietary patterns, such as the Balanced Plate Method. The Balanced Plate Method is likely prominent in culturally specific and linguistically diverse patient resources as it is a visual tool recommending consumption of meals with half a plate of non-starchy vegetables, a quarter of lean proteins, and a quarter of carbohydrate-rich foods that can accommodate traditional foods, flavors, and cooking methods and transcend language barriers. Despite comprehensive inclusion of disease management goals and MNT topics, most culturally specific resources were published before 2017, and one-third of resources with cultural specificity and third-language translations were published before 2011, indicating that their contents may not reflect latest evidence on nutritional management for diabetes, which has evolved over the last decade (20, 38). While RDs may be able to adapt older resources to newer evidence, updating culturally specific and linguistically diverse patient education resources would likely benefit the delivery of evidence-based MNT to culturally diverse individuals.

A strength of this study is its use of systematic search strategies adapted for gray literature, including consultations with RD CDEs across Canada, which provided a comprehensive collection of resources both available and relevant to clinical practice. Limitations include the inability to capture all resources used in clinical practice due to applied exclusion criteria. As a cross-sectional study, resource availability and content may have changed since data collection. This study did not consider aspects other than culturally specific nutrition education resources that are important to culturally competent diabetes care, such as clinicians’ cultural beliefs, social roles, and implicit biases (39) and intervention design (10). Previous work highlights a need for improved readability and quality of diabetes patient education materials (40, 41). However, the effectiveness of resources identified was outside the scope of this study.

To meet the needs of Canada’s ethnoculturally diverse population, professional resources that provide concrete guidance on tailoring specific MNT topics to diverse cultural contexts, alongside more patient resources targeting underrepresented cultural groups, offering third-language translations, and combining cultural specificity with language concordance are likely needed. Future research interested in the cultural and linguistic diversity of nutrition education resources for diabetes care can explore how clinicians and patients perceive current resources to meet therapeutic and cultural needs of MNT and examine and compare the effectiveness of patient education resources with and without cultural specificity and third-language translations.
