# Supplementary material for: Cultural specificity and linguistic diversity in medical nutrition therapy education resources for type 2 diabetes
Source: Front Clin Diabetes Healthc. 2026 May 15;7:1823506. doi: 10.3389/fcdhc.2026.1823506 (PMC13218994; doi:10.3389/fcdhc.2026.1823506)
Supplement: Supplementary file 1 [file DataSheet1.docx]

Supplementary Material

**Table of Contents**

[1 Comorbidity management goals and medical nutrition therapy topics used during data extraction 2](#_Toc227580163)

[2 Documentation of data sampling for professional and patient education resources used in MNT for T2D 4](#_Toc227580164)

[2.1 Bibliographic Databases – Professional Resources 4](#_Toc227580165)

[2.2 Bibliographic Databases – Patient Resources 5](#_Toc227580166)

[2.3 Grey Literature Databases – Professional Resources 6](#_Toc227580167)

[2.4 Grey Literature Databases – Patient Resources 6](#_Toc227580168)

[2.5 Customized Google Search Engine – Professional and Patient Resources 7](#_Toc227580169)

[2.6 Targeted Websites – Professional Resources 8](#_Toc227580170)

[2.7 Targeted Websites – Patient Resources 9](#_Toc227580171)

[2.8 Knowledge Experts – Professional Resources 12](#_Toc227580172)

[2.9 Knowledge Experts – Patient Resources 14](#_Toc227580173)

[3 ACCODS Checklist 19](#_Toc227580174)

[4 Study flow diagram of systematic search of professional and patient education resources 21](#_Toc227580175)

[5 Descriptive characteristics of professional and patient education resources 21](#_Toc227580176)

[5.1 Descriptive characteristics of professional resources 22](#_Toc227580177)

[5.2 Descriptive characteristics of patient resources 29](#_Toc227580178)

[6 Geographic location of knowledge experts (RD CDEs) consulted 56](#_Toc227580179)

[7 Detailed comparison of descriptive characteristics among resources by cultural specificity and language availability 57](#_Toc227580180)

[8 Figure comparing descriptive characteristics among resources by cultural specificity and language availability 59](#_Toc227580181)

# Comorbidity management goals and medical nutrition therapy topics used during data extraction

| Comorbidity management goals | Description |
| --- | --- |
| Blood sugar management | Prevention or management of hyperglycemia, hypoglycemia, DKA, or HHS; effect of nutrition therapy on glycemia. |
| Cardiovascular disease | Prevention or management of CVD or dyslipidemia; effect of nutrition therapy on CVD or dyslipidemia. |
| Weight management | Prevention or management of overweight or obesity; effect of nutrition therapy on weight loss; weight maintenance; achieving best weight. |
| Hypertension | Management of hypertension; effect of nutrition therapy on hypertension. |
| Gastrointestinal health | General digestive health, effect of nutrition interventions on gastrointestinal symptoms (e.g. constipation, diarrhea, gastroesophageal reflux). |
| Gastroparesis | Management of gastroparesis; effect of nutrition therapy on gastroparesis. |
| Nephropathy | Prevention or management of nephropathy; effect of nutrition therapy on nephropathy. |
| Nephropathy | Prevention or management of nephropathy; effect of nutrition therapy on nephropathy. |
| Retinopathy | Prevention or management of retinopathy; effect of nutrition therapy on retinopathy. |
| Mental health | Prevention or management of adverse mental health conditions when undergoing nutrition therapy; effect of nutrition therapy on mental health. |
| MNT topic | Description |
| General healthy eating | Recommendations on food selection for people with T2D without specific details on meal planning or snack planning (e.g. eat more vegetables; choose leaner meats). |
| Meal planning | Recommendations for planning and/or preparing a nutritionally balanced meal and/or appropriate meal timing and/or meal portions a person living with T2D; food preparation, cooking skills, grocery shopping, recipes; meal plans. |
| Snack planning | Recommendations for planning and/or preparing a nutritionally balanced snack; appropriate snack timing, and/or snack portions for a person living with T2D. |
| Macronutrients | Macronutrient distributions, effect of carbohydrates (including sugar, starch, and fiber), fat, or protein on glucose homeostasis or comorbidities; Guidance on consuming specific quantities of macronutrients for people living with T2D. |
| Micronutrients | Micronutrient intake for people with T2D, effect of micronutrients on glucose homeostasis or comorbidities. Guidance on consuming specific quantities of micronutrients for people living with T2D. |
| Carbohydrate counting | Counting the grams of carbohydrate in foods; counting the servings of carbohydrate rich foods at meals or snacks. |
| Label reading | Understanding and interpreting a Nutrition Facts Label, food ingredient list, and/or claims on food labels (e.g. health claims, nutrient content claims, structure or function claims). |
| Eating away from home | Recommendations for food selection, meal planning, or snack planning when consuming food away from home. |
| Non-nutritive sweeteners | Effect of non-nutritive sweeteners on glucose homeostasis or comorbidities; recommendations to consume non-nutritive sweeteners |
| Any dietary pattern | Mention of any dietary pattern listed below |
| Balanced Plate Method | Guidance to consume meals that are structured as half a plate of vegetables, a quarter plate of protein-rich foods, a quarter plate of starchy foods, with or without a fruit and a glass of milk/alternatives or water. |
| Low glycemic index diet | Guidance to follow a dietary pattern rich in low glycemic index foods. |
| Canada’s Food Guide | Guidance to follow the 2007 or 2019 version of Canada’s Food Guide. |
| Mediterranean diet | Guidance to follow a diet high in plant foods like vegetables, fruits, whole grains, nuts and seeds, olive oil and legumes, consume fish, seafood, eggs and low-fat milk and milk products in moderate amounts, and consume meat and meat products and sweet foods occasionally, with or without discussion of consuming wine with meals. Effect of Mediterranean diet on glucose homeostasis or comorbidities. |
| DASH diet | Guidance to follow the “Dietary Approaches to Stop Hypertension” diet (high in vegetables, fruits, whole grains, calcium-rich foods, fish and poultry, legumes and nuts; limited in fatty meats, full-fat dairy, tropical oils; sweets; sodium). Effect of DASH diet on glucose homeostasis or comorbidities. |
| Vegetarian diet | Guidance to follow a lacto-ovo, lacto-, ovo-, or vegan dietary pattern; effect of vegetarian and vegan diets on glucose homeostasis or comorbidities. |
| Low fat diet | Explicit guidance on following a dietary pattern limited in total and/or saturated fat. Effect of a low-fat diet on glucose homeostasis or comorbidities. |
| Low calorie diet | Explicit guidance on following a dietary pattern restricted in total calories. Effect of a low-calorie diet on glucose homeostasis or comorbidities. |
| Intermittent fasting | Guidance on structured fasting for the purpose of weight loss, improving health, and/or wellbeing; effect of structured fasting on glucose homeostasis or comorbidities. Does not include fasting for religious reasons. |
| Portfolio diet | Guidance on following a dietary pattern rich in nuts, plant sterols, fibre, and soy protein (shown to benefit dyslipidemia). Effect of the Portfolio diet on glucose homeostasis or comorbidities. |
| Nordic diet | Guidance on following the Nordic translation of the Mediterranean, Portfolio, DASH and NCEP dietary patterns, using foods typically consumed as part of a traditional Nordic diet in the context of Nordic Nutrition Recommendations. Effect of the Nordic diet on glucose homeostasis or comorbidities. |
| Popular weight loss diets | Effects of the Atkins, Zone, Ornish, Weight Watchers, Protein Power Lifeplan diets on glucose homeostasis or comorbidities. |
| Gluten-free diet | Adapting dietary patterns for the management of T2D with gluten-free food choices; nutritional management of T2D and celiac disease. Effect of gluten-free dietary patterns on glucose homeostasis or comorbidities. |

# Documentation of data sampling for professional and patient education resources used in MNT for T2D

## Bibliographic Databases – Professional Resources

| **Date** | **Database Name** | **Search Strategy (s)** *All results retrieved were reviewed for relevance by 1 reviewer.* | **# of items retrieved** | **# of items included for screening** |
| --- | --- | --- | --- | --- |
| **Professional Resources** | | | | |
| 06-Feb- 2023 | CINAHL | 1) ((MH "Diabetes Mellitus+") OR TI diabet* OR AB diabet*) AND ((MH "Diet+") OR (MH "Nutrition+") OR TI ( diet* or nutrition* ) OR AB ( diet* or nutrition* ) ) AND ((MH "Practice Guidelines") OR TI "practice guideline*" OR AB "practice guideline*" ) AND (MH "Canada+"); published after 2012 | 1) 28 | 1) 2 |
|  | Ovid | 1) (diabetes mellitus/ or diabetes mellitus, type 2/) AND (diet/ or food/ or eating) AND (Publication Type: practice guideline OR practice guidelines as topic/); published after 2012 | 1) 40 | 1) 0 |
|  | Pubmed | 1) (diabet*) AND (food* or nutrition* or diet*) AND (Canad*) AND Limits: practice guideline or guideline AND past 10 years | 1) 6 | 1) 0 |
| 07-Feb-2023 | JBI EDP Database | 1) (diabet* and (nutrition* or food* or diet*)).m_titl. | 1) 10 | 1) 0 |
| **Total items included for screening** | | | | **2** |

## Bibliographic Databases – Patient Resources

| **Date** | **Database Name** | **Search Strategy (s)** *All results retrieved were reviewed for relevance by 1 reviewer.* | **# of items retrieved** | **# of items included for screening** |
| --- | --- | --- | --- | --- |
| **Patient Resources** | | | | |
| 06-Feb-2023 | CINAHL | 1) diabet* N2 (2 OR two OR II) AND ( food* OR diet* ) AND ( "patient* information" OR "patient* education" ) AND Canada+; published after 2012  2) diabet* AND diet* AND [( "patient* information" OR "patient* education" ) OR (MH "Patient Education") OR (MH "Diabetes Education") OR (MH "Nutrition Education") ] AND Canada+; published after 2012  3) (MH "Diabetes Mellitus+/DH") AND [( "patient* information" OR "patient* education" ) OR (MH "Patient Education") OR (MH "Diabetes Education") OR (MH "Nutrition Education") ] AND Canada+; published after 2012 | 1) 24 2) 38 3) 172 | 1) 0 2) 0 3) 0 |
|  | Ovid | 1) (diabetes mellitus/ or diabetes mellitus, type 2/) AND (nutrition therapy/ or diet therapy/) AND (Health Education/); published after 2012  2) (diabetes mellitus/ or diabetes mellitus, type 2/) AND (nutrition therapy/ or diet therapy/) AND Publication Type: Patient Education Handout   3) (diabetes mellitus/ or diabetes mellitus, type 2/) AND (diet/ or food/ or eating) AND Publication Type: Patient Education Handout; published after 2012 | 1) 9 2) 0 3) 1 | 1) 0 2) 0 3) 0 |
|  | Pubmed | 1) (diabet*) AND (food* or nutrition* or diet*); Limits: patient education handout AND 10 years  2) (diabet*) AND (food* or nutrition* or diet*) AND ("patient* education" or "patient* information" or "handout*") AND (Canad*); Limits: patient education handout, books, review, systematic review AND past 10 years | 1) 6 2) 19 | 1) 0 2) 0 |
| 07-Feb-2023 | JBI EDP Database | 1) (diabet* and (nutrition* or food* or diet*)).m_titl. | 1) 10 | 1) 0 |
| **Total items included for screening** | | | | **0** |

## Grey Literature Databases – Professional Resources

| **Date** | **Database Name** | **Search Strategy (s)** *All results retrieved were reviewed for relevance by 1 reviewer.* | **# of items retrieved** | **# of items included for screening** |
| --- | --- | --- | --- | --- |
| **Professional Resources** | | | | |
| 07-Feb-2023 | TRIP Database (free) | 1) diabet* AND (diet* OR nutrition* OR food*) - filters: guidelines, Canada 2) diabet* AND ("medical nutrition")- filters: guidelines, Canada | 1) 175 2) 3 | 1) 1 2) 0 |
|  | BC Guidelines and Protocols | Hand-searched from www2.gov.bc.ca/gov/content/health/practitioner-professional-resources/bc-guidelines/guidelines-by-topic | 1) 1 | 1) 1 |
|  | CPG Infobase | 1) diabet* and (nutrition* OR food* OR diet*) - Limits: adult, elderly, general | 1) 47 | 1) 3 |
|  | Guidelines International Network | 1) diabet* and (nutrition* OR food* OR diet*) 2) diabet* - Limit: Canada | 1) 0 2) 2 | 1) 0 2) 0 |
|  | ClinicalKey | 1) diabet* and (nutrition* OR food* OR diet*) and (canad*) - limit Guidelines | 1) 6 | 1) 0 |
| **Total items included for screening** | | | | **5** |

## Grey Literature Databases – Patient Resources

| **Date** | **Database Name** | **Search Strategy (s)** *The first 100 results retrieved were reviewed for relevance by 1 reviewer.* | **# of items retrieved** | **# of items included for screening** |
| --- | --- | --- | --- | --- |
| **Professional Resources** | | | | |
| 07-Feb-2023 | TRIP Database (free) | "1) diabet* AND (diet* OR nutrition* OR food*) – Limits: patient information leaflets, Canada | 1) 2797  2) 7 | 1) 0 2) 0 |
|  | ClinicalKey | 1) diabet* and (nutrition* OR food* OR diet*) and (canad*) - limit Patient Education | 1) 6 | 1) 1 |
| **Total items included for screening** | | | | **1** |

## Customized Google Search Engine – Professional and Patient Resources

| **Date** | **Database Name** | **Search Strategy (s)** *The first 100 results retrieved were reviewed for relevance by 1 reviewer.* | **# of items retrieved** | **# of items included for screening** | |  |
| --- | --- | --- | --- | --- | --- | --- |
| **Professional and Patient Resources** | | | | | | |
| 21-Mar-2023 | Canadian Public Health Information (CPHI)  www.ophla.ca/p/customsearchcanada.html | 1) diabet* and (nutrition* OR food* OR diet*) and guideline*   Selection: Items were selected by scanning first 100 results for each province: Federal, NCCs, AB, BC, MB, NB, NL, NT, NS, NU, ON, PEI, QC, SK, YT  Sort: Relevance | Federal) 4,640 NCCs) 392 AB) 808 BC) 5,030 MB) 1,020 NB) 922 NL) 101 NT) 305 NS) 814 NU) 109 ON) 3,580 PEI) 393 QC) 1,010 SK) 615 YT) 0 | Federal) 0 NCCs) 2 AB) 9 BC) 10 MB) 8 NB) 0 NL) 0 NT) 3 NS) 3 NU) 4 ON) 2 PEI) 0 QC) 5 SK) 6 YT) 0 | |  |
| **Total items included for screening** | | | | | **37 Professional**  **15 Patient** | |

## Targeted Websites – Professional Resources

| **Date** | **Source Organization and Website Name** | **URL** | **Search Strategy (s)** | **# of items included for screening** |
| --- | --- | --- | --- | --- |
| **Professional Resources** | | | | |
| 06-Feb- 2023 | Diabetes Canada – Clinical Practice Guidelines Website | <https://guidelines.diabetes.ca/cpg> | All websites were hand searched by one reviewer for items of relevance. | 3 |
|  | National Aboriginal Diabetes Association | <https://nada.ca/> |  | 1 |
|  | Alberta Health Services – Nutrition & Disease Management: Nutrition, Information for Health Professionals | <https://www.albertahealthservices.ca/nutrition/Page15593.aspx> |  | 2 |
|  | Government of Nunavut – Healthy Eating | <https://www.pennutrition.com/index.aspx> |  | 1 |
| 07-Feb-2023 | Dietitians of Canada – Practice-Based Evidence in Nutrition (PEN) | <https://www.pennutrition.com/index.aspx> |  | 5 |
|  | Diabetes Canada | <https://www.diabetes.ca/> |  | 0 |
|  | Diabetes Quebec | <https://www.diabete.qc.ca/en/> |  | 0 |
|  | Dietitians of Canada – Unlockfood | <https://www.unlockfood.ca/en/Diabetes.aspx> |  | 0 |
| 21-Mar-2023 | Diabetes Canada – Clinical Practice Guidelines Youtube Page | <https://www.youtube.com/@CDACPG> |  | 2 |
|  | Diabetes Canada –Youtube Page | <https://www.youtube.com/@CDA1927/videos> |  | 4 |
| 23-Mar-2023 | National Collaborating Centre for Indigenous Health (NCCIH) | <https://www.nccih.ca/34/Publication.nccih?cat=15> |  | 4 |
| **Total items included for screening** | | | | **22** |

## Targeted Websites – Patient Resources

| **Date** | **Source Organization and Website Name** | **URL** | **Search Strategy (s)** | **# of items included for screening** |
| --- | --- | --- | --- | --- |
| **Professional Resources** | | | | |
| 06-Feb- 2023 | Diabetes Canada Clinical Practice Guidelines: Patient Resources | <https://guidelines.diabetes.ca/patient-resources> | All websites were hand searched by one reviewer for items of relevance. | 14 |
|  | HealthLinkBC | <https://www.healthlinkbc.ca/> |  | 6 |
|  | Fraser Health | <https://www.fraserhealth.ca/health-topics-a-to-z/diabetes#.Y-HsJHbMJD-> |  | 1 |
|  | National Aboriginal Diabetes Association | <https://nada.ca/> |  | 14 |
|  | First Nations Health Authority | <https://www.fnha.ca/> |  | 0 |
|  | Indigenous Diabetes Health Circle | <https://idhc.life/resources/> |  | 1 |
|  | Health Canada | <https://www.canada.ca/en/public-health/services/diseases/type-2-diabetes.html> |  | 1 |
|  | Alberta Health Services | <https://www.albertahealthservices.ca/nutrition/Page11115.aspx> |  | 6 |
|  | Saskatchewan Health Authority | <https://www.suncountry.sk.ca/service/17/88/dietitian.html> |  | 1 |
|  | Government of Manitoba | <https://www.gov.mb.ca/health/> |  | 0 |
|  | Winnipeg Regional Health Authority | <https://wrha.mb.ca/diabetes-service-guide/resources/> |  | 0 |
|  | Government of New Brunswick | <https://www2.gnb.ca/content/gnb/en/departments/health.html> |  | 0 |
|  | Government of Newfoundland and Labrador | <https://www.gov.nl.ca/hcs/wellnesshealthyliving/> |  | 0 |
|  | Nova Scotia Health | <https://library.nshealth.ca/DiabetesNS/learning> |  | 0 |
|  | Government of Northwestern Territories | <https://www.hss.gov.nt.ca/en/services/diabetes/frequently-asked-questions> |  | 0 |
|  | Government of Ontario | <https://www.ontario.ca/page/preventing-and-living-diabetes#page> |  | 1 |
|  | Health Prince Edward Island (PEI) | <https://www.princeedwardisland.ca/en/topic/health-pei> |  | 0 |
|  | Government of Nunavut – Healthy Eating | <https://livehealthy.gov.nu.ca/en/healthy-eating> |  | 2 |
|  | Government of Yukon | <https://yukon.ca/en/search?query=diabetes> |  | 3 |
| 07-Feb-2023 | Dietitians of Canada – Practice-Based Evidence in Nutrition (PEN) | <https://www.pennutrition.com/index.aspx> |  | 15 |
|  | Diabetes Canada | <https://www.diabetes.ca/> |  | 14 |
|  | Diabetes Quebec | <https://www.diabete.qc.ca/en/> |  | 37 |
|  | Dietitians of Canada – Unlockfood | <https://www.unlockfood.ca/en/Diabetes.aspx> |  | 28 |
| 21-Mar-2023 | Diabetes Canada –Youtube Page | <https://www.youtube.com/@CDA1927/videos> |  | 15 |
| **Total items included for screening** | | | | **159** |

## Knowledge Experts – Professional Resources

| **Date** | **RD CDE id** | **Method of Consult** | **Location** | **Professional Resources** | **# of items included for screening** | **Rationale** |
| --- | --- | --- | --- | --- | --- | --- |
| 2-May-2023 | 1 | **Phone** | **ON** | Diabetes Canada Clinical Practice Guidelines | 63 | N/A |
| 3-May-2023 | 2 | **Phone** | **QC** | Diabetes Canada Clinical Practice Guidelines | 0 | Already included |
| 4-May-2023 | 3 | **Email** | **SK** | Diabetes Canada Clinical Practice Guidelines | 0 | Already included |
| 4-May-2023 | 4 | **Email** | **PEI** | Diabetes Canada Clinical Practice Guidelines | 0 | Already included |
| 4-May-2023 | 5 | **Zoom** | **QC** | Diabetes Canada Clinical Practice Guidelines | 0 | Already included |
|  |  |  |  | Academic conferences (Diabetes Canada, Charles Best) | 0 | Ineligible |
|  |  |  |  | Clinic journal clubs | 0 | Ineligible |
| 4-May-2023 | 6 | **Email** | **AB** | Diabetes Canada Clinical Practice Guidelines | 0 | Already included |
|  |  |  |  | Alberta Health Services Guidelines (Diabetes) | 13 | N/A |
| 8-May-2023 | 7 | **Phone** | **ON** | Diabetes Canada Clinical Practice Guidelines | 0 | Already included |
|  |  |  |  | Canadian Cardiovascular Society Guidelines for the  Management of Dyslipidemia | 2 | N/A |
|  |  |  |  | Alberta Health Services Guidelines (Diabetes) | 0 | Already Included |
| 8-May-2023 | 8 | **Zoom** | **SK & AB** | Diabetes Canada Clinical Practice Guidelines | 0 | Already Included |
|  |  |  |  | Alberta Health Services Guidelines (Diabetes) | 0 | Already included |
|  |  |  |  | Medtronic Pump guidelines | 0 | Ineligible |
| 8-May-2023 | 9 | **Zoom** | **AB** | Diabetes Canada Clinical Practice Guidelines | 0 | Already Included |
|  |  |  |  | Alberta Health Services Guidelines (Diabetes) | 0 | Already included |
| 9-May-2023 | 10 | **Zoom** | **ON** | Diabetes Canada Clinical Practice Guidelines | 0 | Already included |
|  |  |  |  | Academic conferences (Diabetes Canada) | 0 | Ineligible |
| 15-May-2023 | 11 | **Email** | **NU** | Diabetes Canada Clinical Practice Guidelines | 0 | Already included |
| 16-May-2023 | 12 | **Phone** | **NB** | Diabetes Canada Clinical Practice Guidelines | 0 | Already included |
|  |  |  |  | Academic conferences (Provincial diabetes-related) | 0 | Ineligible |
|  |  |  |  | Education sessions provided by industry (e.g. insulin pumps, continuous glucose monitoring devices) | 0 | Ineligible |
| 17-May-2023 | 13 | **Phone** | **MB** | Diabetes Canada Clinical Practice Guidelines | 0 | Already included |
|  |  |  |  | Practice-based Evidence in Nutrition (PEN) | 3 | N/A |
|  |  |  |  | Alberta Health Services Guidelines (Diabetes) | 0 | Already included |
| 17-May-2023 | 14 | **Phone** | **NB** | Diabetes Canada Clinical Practice Guidelines | 0 | Already included |
|  |  |  |  | Canadian Cardiovascular Society Guidelines for the  Management of Dyslipidemia | 0 | Already included |
| 18-May-2023 | 15 | **Zoom** | **NS** | Diabetes Canada Clinical Practice Guidelines | 0 | Already included |
|  |  |  |  | Print resources provided by pharmaceutical companies | 1 | One publicly available, others ineligible |
|  |  |  |  | Education sessions provided by industry | 0 | Ineligible |
|  |  |  |  | Conversation with community cooks and elders | 0 | Ineligible |
|  |  |  |  | Conversation with colleagues (doctors, nurse practitioners) | 0 | Ineligible |
| 23-May-2023 | 16 | **Email** | **NB** | Diabetes Canada Clinical Practice Guidelines | 0 | Already included |
|  |  |  |  | Practice-based Evidence in Nutrition (PEN) | 2 | N/A |
| 25-May-2023 | 17 | **Zoom** | **AB** | Diabetes Canada Clinical Practice Guidelines | 0 | Already included |
|  |  |  |  | Alberta Health Services Guidelines (Diabetes) | 0 | Already included |
| 26-May-2023 | 18 | **Zoom** | **PEI** | Diabetes Canada Clinical Practice Guidelines | 0 | Already included |
| 26-May-2023 | 19 | **Phone** | **ON** | Diabetes Canada Clinical Practice Guidelines | 0 | Already included |
|  |  |  |  | Clinic journal clubs | 0 | Ineligible |
|  |  |  |  | Personal review of literature | 0 | Ineligible |
| 26-May-2023 | 20 | **Zoom** | **QC** | Diabetes Canada Clinical Practice Guidelines | 0 | Already included |
|  |  |  |  | Cree Board of Health and Social Services internal guidelines for diabetes care in indigenous populations | 0 | Ineligible |
| 26-May-2023 | 21 | **Zoom** | **BC** | Diabetes Canada Healthcare Huddle Podcasts – Cultural Competency series | 5 | N/A |
|  |  |  |  | First Nations Health Authority “Setting the Table for a Healthy Food Conversation” Handouts | 2 | N/A |
| 29-May-2023 | 22 | **Phone** | **BC** | Diabetes Canada Clinical Practice Guidelines | 0 | Already included |
|  |  |  |  | Obesity Canada Clinical Practice Guidelines | 20 | N/A |
|  |  |  |  | Kidney Disease: Improving Global Outcomes (KDIGO) guidelines in chronic kidney disease | 2 | N/A |
| 30-May-2023 | 23 | **Zoom** | **QC** | Diabetes Canada Clinical Practice Guidelines | 0 | Already included |
|  |  |  |  | American Diabetes Association Practice Guidelines | 0 | Ineligible |
| 05-Jun-2023 | 24 | **Zoom** | **BC** | Diabetes Canada Clinical Practice Guidelines | 0 | Already included |
|  |  |  |  | Conversation with community members and elders | 0 | Ineligible |
|  |  |  |  | Truth and Reconciliation Commission Reports | 0 | Ineligible |
|  |  |  |  | Cultural safety and humility training | 0 | Ineligible |
|  |  |  |  | Diabetes Canada Healthcare Huddle Podcasts – Cultural Competency series | 0 | Already included |
| 06-Jun-2023 | 25 | **Phone** | **BC** | Diabetes Canada Clinical Practice Guidelines | 0 | Already included |
|  |  |  |  | American Diabetes Association Practice Guidelines | 0 | Ineligible |
|  |  |  |  | Practice-based Evidence in Nutrition | 0 | Already included |
|  |  |  |  | Personal review of literature | 0 | Ineligible |
|  |  |  |  | Online conferences and webinars | 0 | Ineligible |
|  |  |  |  | Education sessions provided by industry | 0 | Ineligible |
|  |  |  |  | Conversation with colleagues (doctors, nurse practitioners) | 0 | Ineligible |
| 06-Jun-2023 | 26 | **Phone** | **NL** | Diabetes Canada Clinical Practice Guidelines | 0 | Already included |
|  |  |  |  | Clinic journal clubs | 0 | Ineligible |
|  |  |  |  | Education sessions provided by industry | 0 | Ineligible |
| **Total # of professional resources included** | | | | | **111** |  |

## Knowledge Experts – Patient Resources

| **Date** | **RD CDE id** | **Method of Consult** | **Location** | **Patient Resources** | **# of items included for screening** | **Rationale for exclusion** |
| --- | --- | --- | --- | --- | --- | --- |
| 2-May-2023 | 1 | **Phone** | **ON** | Diabetes Canada handouts | 38 | N/A |
|  |  |  |  | Ethnomed handouts | 0 | Ineligible |
|  |  |  |  | Community Health Centre (CHC) RD Ontario Action Group Resources | 0 | Ineligible |
|  |  |  |  | Diabetes Food Guide (Centretown Community Health) | 1 | N/A |
|  |  |  |  | Alberta Health Services handouts | 12 | N/A |
|  |  |  |  | Hamilton Health handouts | 20 | N/A |
| 3-May-2023 | 2 | **Phone** | **QC** | Type1Better handouts (some applicable for type 2) | 6 | N/A |
|  |  |  |  | Institut de recherches cliniques de Montréal (IRCM) internal handouts | 0 | Ineligible |
| 4-May-2023 | 3 | **Email** | **SK** | Diabetes Canada handouts | 0 | Already included |
| 4-May-2023 | 4 | **Email** | **PEI** | Diabetes Canada handouts | 0 | Already included |
|  |  |  |  | Health Canada – Label Reading handout | 1 | N/A |
|  |  |  |  | Internally developed handouts | 0 | Ineligible |
| 4-May-2023 | 5 | **Zoom** | **QC** | Diabetes Canada handouts | 0 | Already included |
|  |  |  |  | LMC Endocrinology and Diabetes handouts | 26 | N/A |
|  |  |  |  | Type1Better handouts | 0 | Already included |
| 4-May-2023 | 6 | **Email** | **AB** | Alberta Health handouts | 0 | Already included |
| 8-May-2023 | 7 | **Phone** | **ON** | Diabetes Canada handouts | 0 | Already included |
|  |  |  |  | University Health Network – Diabetes College website articles and handouts | 27 | N/A |
|  |  |  |  | PEN handouts | 20 | N/A |
|  |  |  |  | Unlockfood.ca – Diabetes articles | 27 | N/A |
|  |  |  |  | Alberta Health Services handouts | 0 | Already included |
|  |  |  |  | Guelph family health study handouts | 3 | N/A |
| 8-May-2023 | 8 | **Zoom** | **SK & AB** | Diabetes Canada handouts | 0 | Already included |
|  |  |  |  | Patient support group discussions | 0 | Ineligible |
| 8-May-2023 | 9 | **Zoom** | **AB** | Diabetes Canada handouts | 0 | Already included |
|  |  |  |  | Alberta Health Services handouts | 0 | Already included |
| 9-May-2023 | 10 | **Zoom** | **ON** | Diabetes Canada handouts | 0 | Already included |
|  |  |  |  | Health Canada – Label Reading handout | 0 | Already included |
|  |  |  |  | Diabetes Food Guide (Centretown Community Health) | 0 | Already included |
| 15-May-2023 | 11 | **Email** | **NU** | Diabetes Canada handouts | 0 | Already included |
|  |  |  |  | Nunavut Food Guide | 1 | N/A |
|  |  |  |  | Organization-specific handouts (internal access only) | 0 | Ineligible |
| 16-May-2023 | 12 | **Phone** | **NB** | Diabetes Canada handouts | 0 | Already included |
|  |  |  |  | Health Canada – Label Reading handout | 0 | Already included |
|  |  |  |  | Smartphone applications (e.g. MyFitnessPal, Calorie King) | 0 | Ineligible |
|  |  |  |  | Food labels for demonstration of label reading | 0 | Ineligible |
| 17-May-2023 | 13 | **Phone** | **MB** | Diabetes Canada handouts | 0 | Already included |
|  |  |  |  | PEN handouts | 0 | Already included |
|  |  |  |  | Unlockfood.ca – Diabetes articles | 0 | Already included |
| 17-May-2023 | 14 | **Phone** | **NB** | Diabetes Canada handouts | 0 | Already included |
|  |  |  |  | PEN handouts | 0 | Already Included |
|  |  |  |  | Smartphone applications (e.g. MyFitnessPal, Calorie King) | 0 | Ineligible |
|  |  |  |  | Organization-specific handouts (internal access only) | 0 | Ineligible |
|  |  |  |  | Websites containing recipes (not diabetes specific) | 0 | Ineligible |
|  |  |  |  | Health Canada – Nutrient Value of Some Common Foods | 1 | N/A |
| 18-May-2023 | 15 | **Zoom** | **NS** | Diabetes Canada handouts | 0 | Already included |
|  |  |  |  | Organization-specific handouts (internal access only) | 0 | Ineligible |
|  |  |  |  | Websites containing recipes (not diabetes specific) | 0 | Ineligible |
|  |  |  |  | Group education workshops | 0 | Ineligible |
|  |  |  |  | Sharing circles facilitated by an elder | 0 | Ineligible |
| 23-May-2023 | 16 | **Email** | **NB** | Diabetes Canada handouts | 0 | Already included |
|  |  |  |  | Diabetes Canada website articles | 4 | N/A |
|  |  |  |  | Physical food models | 0 | Ineligible |
|  |  |  |  | Unlockfood.ca – diabetes articles | 0 | Already included |
|  |  |  |  | PEN handouts | 0 | Already included |
| 25-May-2023 | 17 | **Zoom** | **AB** | Alberta Health Services handouts | 0 | Already included |
|  |  |  |  | Diabetes Canada handouts | 0 | Already included |
|  |  |  |  | Diabetes Australia handouts | 0 | Ineligible |
|  |  |  |  | Ethnomed handouts | 0 | Ineligible |
|  |  |  |  | Alberta Diabetes Link handouts | 12 | N/A |
|  |  |  |  | Local resources for food aid or assistance | 0 | Ineligible |
| 26-May-2023 | 18 | **Zoom** | **PEI** | Diabetes Canada handouts | 0 | Already included |
|  |  |  |  | Diabetes Food Guide (Centretown Community Health) | 0 | Already included |
|  |  |  |  | Organization-specific handouts (internal access only) | 0 | Ineligible |
| 26-May-2023 | 19 | **Phone** | **ON** | Diabetes Canada handouts | 0 | Already included |
|  |  |  |  | Unlockfood.ca – diabetes articles | 0 | Already included |
|  |  |  |  | PEN Handouts | 0 | Already included |
|  |  |  |  | Nova Scotia Health handouts | 4 | N/A |
|  |  |  |  | Alberta Health Services handouts | 0 | Already included |
|  |  |  |  | Vancouver Coastal Health handouts | 1^[[1]](#footnote-1)^ | N/A |
|  |  |  |  | American Diabetes Association handouts | 0 | Ineligible |
| 26-May-2023 | 20 | **Zoom** | **QC** | Diabetes Canada handouts | 0 | Already included |
|  |  |  |  | Health Canada – Label Reading handout | 0 | Already included |
|  |  |  |  | PEN handouts | 0 | Already included |
|  |  |  |  | Cree Board of Health and Social Services handouts | 3 | N/A |
| 26-May-2023 | 21 | **Zoom** | **BC** | Indigenous Diabetes Health Circle handouts | 3 | N/A |
|  |  |  |  | National Indigenous Diabetes Association handouts | 14 | N/A |
|  |  |  |  | Indigenous Sport, Physical Activity & Recreation Council handouts | 7 | N/A |
|  |  |  |  | Island Health handouts | 8 | N/A |
| 29-May-2023 | 22 | **Phone** | **BC** | British Columbia Renal Agency handouts | 2 | N/A |
|  |  |  |  | Vancouver Coastal Health handouts | 0 | Already included |
| 30-May-2023 | 23 | **Zoom** | **QC** | Diabetes Canada handouts | 0 | Already included |
|  |  |  |  | Diabetes Quebec handouts | 6 | N/A |
|  |  |  |  | Canadian Diabetes Association handouts (prior name of Diabetes Canada; older handouts available in multiple languages) | 9 | N/A |
|  |  |  |  | LMC Endocrinology and Diabetes handouts | 0 | Already included |
|  |  |  |  | Organization-specific handouts (internal access only) | 0 | Ineligible |
| 05-Jun-2023 | 24 | **Zoom** | **BC** | Cree Board of Health and Social Services handouts | 0 | Already Included |
|  |  |  |  | National Indigenous Diabetes Association handouts | 0 | Already included |
|  |  |  |  | Community discussion and story telling | 0 | Ineligible |
| 06-Jun-2023 | 25 | **Phone** | **BC** | Diabetes Canada handouts | 0 | Already included |
|  |  |  |  | Fraser Health handouts | 9 | N/A |
|  |  |  |  | Organization-specific handouts (internal access only) | 0 | Ineligible |
| 06-Jun-2023 | 26 | **Phone** | **NL** | Diabetes Canada handouts | 0 | Already included |
|  |  |  |  | PEN handouts | 0 | Already included |
|  |  |  |  | Vancouver Coastal Health handouts | 0 | Already included |
|  |  |  |  | Hamilton Health handouts | 0 | Already included |
|  |  |  |  | Alberta Health Services handouts | 0 | Already included |
| **Total # of patient resources included** | | | | | **265** |  |

# ACCODS Checklist

The AACODS checklist is designed to enable evaluation and critical appraisal of grey literature.

The Fourth International Conference on Grey Literature held in Washington, DC, in October 1999 defined grey literature as: "that which is produced on all levels of government, academics, business and industry in print and electronic formats, but which is not controlled by commercial publishers."

Grey literature includes theses or dissertations (reviewed by examiners who are subject specialists); conference papers (often peer-reviewed or presented by those with specialist knowledge) and various types of reports from those working in the field. All of these fall into the “expert opinion”

Critical appraisal is “the process of carefully and systematically examining research to judge its trustworthiness, and its relevance and value in a particular context” (Burls 2009)

Grey (unpublished) studies and RCTs should be appraised using the same tools as their black (published) counterparts.

| **ACCODS Checklist** | | **Yes** | **No** | **?** |
| --- | --- | --- | --- | --- |
| **Authority** | Identifying who is responsible for the intellectual content.  Individual author:   - - Associated with a reputable organization?   - Professional qualifications or considerable experience?   - Produced/published other work (grey/black) in the field?   - Recognized expert, identified in other sources?   - Cited by others? (use Google Scholar as a quick check)   - Higher degree student under “expert” supervision?   Organization or group:   - - Is the organization reputable? (e.g. W.H.O)   - Is the organization an authority in the field?   In all cases:   - - Does the item have a detailed reference list or bibliography? |  |  |  |
| **Accuracy** | Does the item have a clearly stated aim or brief?   - - Is so, is this met?   - Does it have a stated methodology?   - If so, is it adhered to?   - Has it been peer-reviewed?   - Has it been edited by a reputable authority?   - Supported by authoritative, documented references or credible sources?   - Is it representative of work in the field?   - If No, is it a valid counterbalance?   - Is any data collection explicit and appropriate for the research?   - If the item is secondary material (e.g. a policy brief of a technical report) refer to the original. Is it an accurate, unbiased interpretation or analysis? |  |  |  |
| **Coverage** | All items have parameters which define their content coverage. These limits might mean that a work refers to a particular population group, or that it excludes certain types of publication. A report could be designed to answer a particular question or be based on statistics from a particular survey.   - - Are any limits clearly stated? |  |  |  |
| **Objectivity** | It is important to identify bias, particularly if it is unstated or unacknowledged.   - - Opinion, expert or otherwise, is still opinion: is the author’s standpoint clear?   - Does the work seem to be balanced in presentation? |  |  |  |
| **Date** | For the item to inform your research, it needs to have a date that confirms relevance.   - - Does the item have a clearly stated date related to content? No easily discernible date is a strong concern.   - If no date is given, but can be closely ascertained, is there a valid reason for its absence?   - Check the bibliography: have key contemporary material been included? |  |  |  |
| **Significance** | This is a value judgment of the item, in the context of the relevant research area.   - - Is the item meaningful? (this incorporates feasibility, utility and relevance)   - Does it add context?   - Does it enrich or add something unique to the research?   - Does it strengthen or refute a current position?   - Would the research area be lesser without it?   - Is it integral, representative, typical?   - Does it have impact? (in the sense of influencing the work or behaviour of others) |  |  |  |

Source: Tyndall J. *ACCODS Checklist.*, <http://dspace.flinders.edu.au/dspace/> (2010).

#
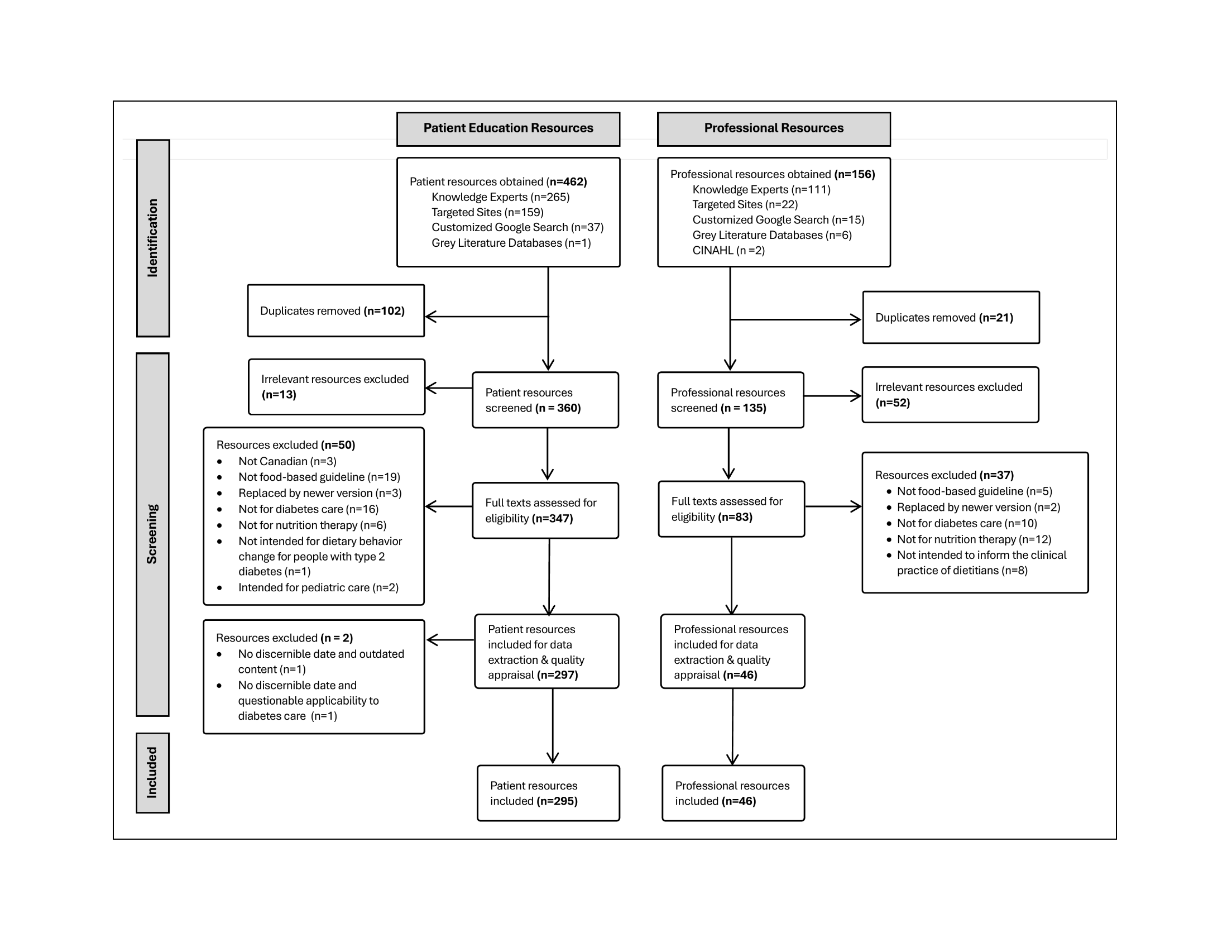
Study flow diagram of systematic search of professional and patient education resources

# Descriptive characteristics of professional and patient education resources

## Descriptive characteristics of professional resources

| # | Source Org. | Year | Title | Type of Org. | Type of Resource | Trans- lations | Specificity to Cultural Groups | Comorbidity Management Goals | Medical Nutrition Therapy Topic |
| --- | --- | --- | --- | --- | --- | --- | --- | --- | --- |
| 1 | Alberta Health Services | 2019 | Nutrition Guideline: Restricted Carbohydrate in Chronic Disease Management | P/T gov. | CPG | None | No | Blood sugar management Cardiovascular disease Weight management Hypertension Nephropathy | Low carbohydrate diet Macronutrients Micronutrients |
| 2 | Alberta Health Services | 2020 | Nutrition Guideline: Sugar Substitutes | P/T gov. | CPG | None | No | Blood sugar management Cardiovascular disease  Hypertension Nephropathy  Mental health Weight management | Non-nutritive sweeteners Macronutrients |
| 3 | Alberta Health Services | 2022 | Nutrition Guideline: Adult Diabetes | P/T gov. | CPG | None | No | Blood sugar management Cardiovascular disease  Hypertension Mental health Weight management Nephropathy | Meal planning Snack planning Glycemic Index Non-nutritive sweeteners Macronutrients Low carbohydrate diet Balanced plate method Canada's Food Guide DASH diet Intermittent fasting Mediterranean diet Vegetarian diet Macronutrients Micronutrients Meal planning Carbohydrate counting Eating away from home Label reading |
| 4 | Alberta Health Services | 2023 | Nutrition Guideline: Household Food Insecurity | P/T gov. | CPG | None | No | Blood sugar management Hypertension Nephropathy  Mental health | Macronutrients Micronutrients Meal planning |
| 5 | AstraZeneca | 2020 | Do You Have Patients with Type 2 Diabetes Who Plan to Fast During Ramadan? | National NGO | Handout | None | Muslin (Ramadan) | Blood sugar management  Nephropathy | Glycemic index Macronutrients Meal planning |
| 6 | Canadian Cardiovascular Society | 2016 | 2016 Canadian Cardiovascular Society Guidelines for the Management of Dyslipidemia for the Prevention of Cardiovascular Disease in the Adult | National NGO | CPG | None | No | Blood sugar management Cardiovascular disease Weight management Hypertension Mental health | DASH diet Low fat diet Glycemic index Mediterranean diet Portfolio diet Vegetarian diet Macronutrients |
| 7 | Canadian Cardiovascular Society | 2021 | 2021 Canadian Cardiovascular Society Guidelines for the Management of Dyslipidemia for the Prevention of Cardiovascular Disease in Adults | National NGO | CPG | None | No | Blood sugar management  Cardiovascular disease Weight management Hypertension Mental health | DASH diet Glycemic index Mediterranean diet Portfolio diet Vegetarian diet Macronutrients |
| 8 | Diabetes Canada | 2017 | Ramadan and Diabetes for Health-Care Professionals | National NGO | Audiovisual | None | Muslin (Ramadan) | Blood sugar management Nephropathy | Balanced plate method Glycemic index Macronutrients Meal planning Snack planning |
| 9 | Diabetes Canada | 2018 | Cardiovascular Protection in People with Diabetes | National NGO | CPG | French | No | Blood sugar management Cardiovascular disease Weight management Hypertension Mental health | Mediterranean diet |
| 10 | Diabetes Canada | 2018 | Chapter 11 Nutrition Therapy | National NGO | Audiovisual | None | Muslin (Ramadan) | Blood sugar management Cardiovascular disease  Hypertension  Weight management | Canada's Food Guide Glycemic index Mediterranean diet Vegetarian diet DASH diet Portfolio diet Macronutrients Micronutrients Meal planning Label reading |
| 11 | Diabetes Canada | 2018 | Chapter 17 Weight Management | National NGO | Audiovisual | None | No | Blood sugar management Cardiovascular disease  Hypertension Weight management | Macronutrients |
| 12 | Diabetes Canada | 2018 | Chronic Kidney Disease in Diabetes | National NGO | CPG | French | No | Blood sugar management Cardiovascular disease Nephropathy | Micronutrients |
| 13 | Diabetes Canada | 2018 | Diabetes and Mental Health | National NGO | CPG | French | No | Blood sugar management Mental health Weight management | Macronutrients |
| 14 | Diabetes Canada | 2018 | Diabetes in Older People | National NGO | CPG | French | No | Blood sugar management Cardiovascular disease Weight management | Macronutrients |
| 15 | Diabetes Canada | 2018 | Dyslipidemia | National NGO | CPG | French | No | Blood sugar management Cardiovascular disease  Weight management | Macronutrients General healthy eating |
| 16 | Diabetes Canada | 2018 | Hypoglycemia | National NGO | CPG | French | No | Blood sugar management Gastroparesis  Weight management | Macronutrients Snack planning |
| 17 | Diabetes Canada | 2018 | Nutrition Therapy | National NGO | CPG | French | African, East Asian, Latin American, Middle Eastern, Muslim (Ramadan), South & Southeast Asian | Blood sugar management Cardiovascular disease  Hypertension Nephropathy  Neuropathy Retinopathy Mental health Weight management | Low carbohydrate diet Canada's Food Guide DASH diet Intermittent fasting Low fat diet Glycemic index Mediterranean diet Nordic diet Popular weight loss diets Portfolio diet Vegetarian diet Macronutrients Micronutrients Meal planning Snack planning Carbohydrate counting Non-nutritive sweeteners |
| 18 | Diabetes Canada | 2018 | Treatment of Hypertension | National NGO | CPG | French | No | Blood sugar management  Weight management Hypertension | Micronutrients General healthy eating |
| 19 | Diabetes Canada | 2018 | Type 2 Diabetes and Indigenous Peoples | National NGO | CPG | French | Indigenous | Blood sugar management Cardiovascular disease  Hypertension Nephropathy  Mental health Weight management | General healthy eating |
| 20 | Diabetes Canada | 2018 | Weight Management in Diabetes | National NGO | CPG | French | No | Blood sugar management Cardiovascular disease  Hypertension  Mental health Weight management | Macronutrients Meal planning |
| 21 | Diabetes Canada | 2019 | Diabetes Canada Position Statement for People with Types 1 and 2 Diabetes Who Fast During Ramadan | National NGO | Position statement | None | Middle Eastern, Muslim (Ramadan), South & Southeast Asian | Blood sugar management Weight management | Glycemic index Macronutrients Meal planning |
| 22 | Diabetes Canada | 2020 | Diabetes Canada Position Statement on Low-Carbohydrate Diets for Adults with Diabetes: A Rapid Review | National NGO | Position statement | None | No | Blood sugar management Cardiovascular disease  Weight management | Low carbohydrate diet Canada's Food Guide Low fat diet Macronutrients Micronutrients |
| 23 | Diabetes Canada | 2020 | Diabetes Canada's Position on Low Carb Diets Webinar | National NGO | Audiovisual | None | No | Blood sugar management Cardiovascular disease Weight management | Low carbohydrate diet Canada's Food Guide DASH diet Intermittent fasting Low fat diet Glycemic index Mediterranean diet Popular weight loss diets Vegetarian diet Macronutrients Micronutrients |
| 24 | Diabetes Canada | 2022 | Cultural Competency Series - Indigenous Communities with Rebecca Sovdi and Rachel Dickens | National NGO | Audiovisual | None | Indigenous | Blood sugar management Mental health | General healthy eating |
| 25 | Diabetes Canada | 2022 | Cultural Competency Series - The Chinese Community with Rosalie Lung | National NGO | Audiovisual | None | East Asian | Blood sugar management  Weight management | Balanced plate method Glycemic index Macronutrients Micronutrients Meal planning Snack planning |
| 26 | Diabetes Canada | 2022 | Cultural Competency Series - The South Asian Community with Jyotika Desai and Siva Swaminathan | National NGO | Audiovisual | None | South & Southeast Asian | Blood sugar management | Macronutrients |
| 27 | Diabetes Canada | 2022 | Dietary Patterns in T1D and T2D with Jenna Walsh | National NGO | Audiovisual | None | No | Blood sugar management Cardiovascular disease Mental health | Low carbohydrate diet Canada's Food Guide DASH diet  Intermittent fasting Mediterranean diet Macronutrients Micronutrients Carbohydrate counting |
| 28 | Diabetes Canada | 2022 | Remission of Type 2 Diabetes | National NGO | CPG | None | No | Blood sugar management  Cardiovascular disease Nephropathy Mental health Weight management Diabetes remission | Low carbohydrate diet Low calorie diet Low fat diet Glycemic index Macronutrients Dietary self-monitoring |
| 29 | Diabetes Canada | 2022 | Remission of Type 2 Diabetes: User's Guide | National NGO | CPG | None | No | Blood sugar management Cardiovascular disease  Hypertension Nephropathy Mental health Weight management Diabetes remission | Low carbohydrate diet Low calorie diet Macronutrients |
| 30 | Government of Nunavut | 2012 | Nunavut Food Guide: Educator's Handbook | P/T gov. | Handout | None | Indigenous | Blood sugar management  Cardiovascular disease | Canada's Food Guide Macronutrients Micronutrients Meal planning Dietary self-monitoring |
| 31 | International Diabetes Federation, Diabetes and Ramadan International Alliance | 2021 | Chapter 8: The Ramadan Nutrition Plan (RNP) for people with diabetes | National NGO | CPG | None | Muslim (Ramadan) | Blood sugar management  Cardiovascular disease  Hypertension Nephropathy Weight management | Balanced plate method Glycemic index Macronutrients Meal planning Snack planning Non-nutritive sweeteners |
| 32 | Island Health | 2013 | Setting the Table: Enjoying, Exploring & Sharing Food | P/T gov. | Handout | None | Indigenous | Blood sugar management | Macronutrients Micronutrients Meal planning Snack planning |
| 33 | Island Health | 2021 | Setting the Table for Safer Food Conversations: The importance of critical self reflection when working with Indigenous Peoples | P/T gov. | Handout | None | Indigenous | Blood sugar management Mental health | Meal planning General healthy eating |
| 34 | National Collaborating Centre for Indigenous Health | 2011 | Traditional Aboriginal Diets and Health | National NGO | Handout | French | Indigenous | Blood sugar management  Cardiovascular disease  Mental health Weight management | Low carbohydrate diet Canada's Food Guide Macronutrients Micronutrients |
| 35 | National Collaborating Centre for Indigenous Health | 2013 | Nutrition Fact Sheet | National NGO | Handout | French | Indigenous | Blood sugar management  Cardiovascular disease Hypertension  Mental health Weight management | Macronutrients Micronutrients Meal planning Label reading |
| 36 | Northern Health | 2019 | Guidelines for Health Professionals: Household Food Insecurity | P/T gov. | CPG | None | Indigenous | Blood sugar management  Cardiovascular disease  Hypertension Mental health | General healthy eating |
| 37 | Obesity Canada | 2020 | Commercial Products and Programs in Obesity Management | National NGO | CPG | None | No | Blood sugar management Cardiovascular disease Hypertension  Mental health Weight management | Low carbohydrate diet Low calorie diet Popular weight loss diets Macronutrients Micronutrients Dietary self-monitoring |
| 38 | Obesity Canada | 2020 | Effective Psychological and Behavioural Interventions in Obesity Management | National NGO | CPG | None | No | Blood sugar management Cardiovascular disease Hypertension  Mental health Weight management | Low carbohydrate diet Intermittent fasting Low calorie diet Mediterranean diet Dietary self-monitoring |
| 39 | Obesity Canada | 2020 | Obesity Management and Indigenous Peoples | National NGO | CPG | None | Indigenous | Blood sugar management Mental health Weight management | General healthy eating Label reading |
| 40 | Obesity Canada | 2022 | Medical Nutrition Therapy in Obesity Management | National NGO | CPG | None | No | Blood sugar management Cardiovascular disease  Hypertension Nephropathy Neuropathy Retinopathy Mental health Weight management Diabetes Remission | Low carbohydrate diet Canada's Food Guide DASH diet Intermittent fasting Low calorie diet Low fat diet Glycemic index Mediterranean diet Nordic diet Portfolio diet Popular weight loss diets Vegetarian diet Macronutrients Micronutrients Label reading Non-nutritive sweeteners |
| 41 | Dietitians of Canada - Practice-based Evidence in Nutrition (PEN) | 2021 | Diet Composition - Ketogenic Diet | National NGO | Report | None | No | Blood sugar management Cardiovascular disease Hypertension  Mental health Weight management Diabetes remission | Low carbohydrate diet Low fat diet Macronutrients Micronutrients |
| 42 | Dietitians of Canada - Practice-based Evidence in Nutrition (PEN) | 2022 | Diet Composition - Mediterranean Diet | National NGO | Report | None | No | Blood sugar management Cardiovascular disease  Hypertension Weight management | DASH diet Low fat diet Mediterranean diet Macronutrients Micronutrients |
| 43 | Dietitians of Canada - Practice-based Evidence in Nutrition (PEN) | 2023 | Diabetes - Glycemic Index | National NGO | Report | None | No | Blood sugar management Cardiovascular disease Gastrointestinal health  Nephropathy Neuropathy Retinopathy Mental health Weight management Diabtes remission | Low carbohydrate diet Low calorie diet Low fat diet Glycemic index Mediterranean diet Macronutrients Micronutrients Meal planning Snack planning Dietary self-monitoring Eating away from home Label reading Non-nutritive sweeteners |
| 44 | Dietitians of Canada - Practice-based Evidence in Nutrition (PEN) | 2023 | Diabetes/ Glucose Intolerance | National NGO | Report | None | No | Blood sugar management Cardiovascular disease  Hypertension  Nephropathy Weight management Diabetes remission | Low carbohydrate diet Low calorie diet Low fat diet Glycemic index Mediterranean diet Vegetarian diet Macronutrients Micronutrients Meal planning Snack planning |
| 45 | Waterloo Wellington Diabetes | 2021 | Therapeutic Carbohydrate Restriction (or Reduction) & Diabetes: A Guide for Health Care Professionals | P/T NGO | Report | None | No | Blood sugar management Cardiovascular disease Hypertension  Nephropathy  Mental health Weight management Diabetes remission | Low carbohydrate diet Intermittent fasting Low calorie diet Macronutrients Micronutrients Carbohydrate counting Label reading Non-nutritive sweeteners |
| 46 | Winnipeg Regional Health Authority | N/A | Diabetes & Eating | P/T gov. | Handout | None | No | Blood sugar management Cardiovascular disease Hypertension Mental health Weight management | Low carbohydrate diet Balanced plate method Canada's Food Guide DASH diet Mediterranean diet Popular weight loss diets Macronutrients Micronutrients Meal planning Carbohydrate counting Glycemic index Label reading Non-nutritive sweeteners |

## Descriptive characteristics of patient resources

| # | Source Org. | Year | Title | Type of Org. | Type of Resource | Translations | Specificity to Cultural Groups | Disease Management Goals | Medical Nutrition Therapy Topics |
| --- | --- | --- | --- | --- | --- | --- | --- | --- | --- |
| 1 | Alberta Diabetes Link | 2016 | Heart Healthy Label Reading | P/T NGO | Handout | None | No | Blood sugar management Cardiovascular disease | Label reading Macronutrients General healthy eating |
| 2 | Alberta Diabetes Link | 2016 | Heart Healthy Eating | P/T NGO | Handout | None | No | Blood sugar management Cardiovascular disease Weight management | Label reading Macronutrients General healthy eating |
| 3 | Alberta Diabetes Link | 2018 | Healthy Eating for Weight and Diabetes Management | P/T NGO | Handout | Arabic; French; Punjabi; Spanish; Tagalog; Urdu | No | Blood sugar management Weight management | Balanced plate method Glycemic index Label reading Micronutrients Meal planning Snack planning |
| 4 | Alberta Diabetes Link | 2016 | Dietary Approach to Stop Hypertension (DASH) and Mediterranean Diet | P/T NGO | Handout | None | No | Blood sugar management Weight management Cardiovascular disease Hypertension | Mediterranean diet DASH diet Balanced plate method Label reading Macronutrients Micronutrients |
| 5 | Alberta Diabetes Link | 2018 | The Glycemic Index | P/T NGO | Handout | None | No | Blood sugar management Cardiovascular disease Weight management | Glycemic index Macronutrients |
| 6 | Alberta Diabetes Link | 2018 | Facts on Dietary Fiber | P/T NGO | Handout | None | No | Blood sugar management Cardiovascular disease Weight management Gastrointestinal health | Label reading Macronutrients General healthy eating |
| 7 | Alberta Diabetes Link | 2018 | Carbohydrate Food List | P/T NGO | Handout | None | No | Blood sugar management | Carbohydrate counting Macronutrients |
| 8 | Alberta Diabetes Link | 2016 | Carbohydrate and Non-Carbohydrate Foods - South Asian | P/T NGO | Handout | None | South & Southeast Asian | Blood sugar management Weight management | Carbohydrate counting Macronutrients |
| 9 | Alberta Diabetes Link | 2018 | Carbohydrate and Non-Carbohydrate Foods | P/T NGO | Handout | Arabic; French; Punjabi; Spanish; Tagalog; Urdu | No | Blood sugar management Weight management | Balanced plate method Carbohydrate counting Macronutrients |
| 10 | Alberta Health Services | 2022 | Learning About Carbohydrate (Carb) Counting and Eating Out When You Have Diabetes | P/T gov. | Web article | None | No | Blood sugar management Weight management | Canada's Food Guide Carbohydrate counting Eating away from home Macronutrients Meal planning |
| 11 | Alberta Health Services | 2022 | Diabetic Renal Diet: Care Instructions | P/T gov. | Web article | None | No | Blood sugar management Nephropathy | Vegetarian diet Label reading Macronutrients Micronutrients Meal planning |
| 12 | Alberta Health Services | 2020 | Sample Meal Plans for Healthy Eating with Diabetes | P/T gov. | Handout | None | No | Blood sugar management | Macronutrients Meal planning Snack planning |
| 13 | Alberta Health Services | 2017 | Healthy Meals to Help Your Diabetes | P/T gov. | Handout | None | No | Blood sugar management | Balanced plate method Macronutrients |
| 14 | Alberta Health Services | 2022 | Learning About Meal Planning for Diabetes | P/T gov. | Web article | None | No | Blood sugar management Weight management | Canada's Food Guide Label reading Eating away from home Macronutrients Meal planning Snack planning Carbohydrate counting |
| 15 | Alberta Health Services | 2017 | Carbohydrate in Foods | P/T gov. | Handout | None | No | Blood sugar management | Carbohydrate counting Macronutrients |
| 16 | Alberta Health Services | 2018 | Healthy Snacks to Help Your Diabetes | P/T gov. | Handout | None | No | Blood sugar management | Macronutrients Snack planning |
| 17 | Alberta Health Services | 2021 | Healthy Eating for People with Diabetes and Kidney DIsease | P/T gov. | Handout | None | No | Blood sugar management Nephropathy | Balanced plate method Carbohydrate counting Label reading Macronutrients Micronutrients Meal planning Snack planning |
| 18 | Alberta Health Services | 2015 | Healthy Eating for Diabetes | P/T gov. | Handout | None | No | Blood sugar management Cardiovascular disease Weight management Gastrointestinal health | Balanced plate method Label reading Non-nutritive sweeteners Macronutrients Micronutrients Meal planning Snack planning |
| 19 | Alberta Health Services | 2018 | Eating When You Have Gastroparesis and Diabetes | P/T gov. | Handout | None | No | Blood sugar management Gastrointestinal health Gastroparesis Nephropathy | Canada's Food Guide Macronutrients Micronutrients |
| 20 | Alberta Health Services | 2017 | Diabetes: Healthy Meal Planning | P/T gov. | Handout | None | No | Blood sugar management | Balanced plate method Carbohydrate counting Label reading Non-nutritive sweeteners  Macronutrients Micronutrients Meal planning Snack planning |
| 21 | Alberta Health Services, Healthwise | 2022 | Diabetes: Using a Plate Format to Plan Meals | P/T NGO | Web article | None | No | Blood sugar management | Canada's Food Guide Balanced plate method Carbohydrate counting Meal planning Snack planning |
| 22 | BC Renal Agency | 2013 | Diabetes and Kidney Diet Basics | P/T gov. | Handout | None | No | Blood sugar management Nephropathy | Label reading Non-nutritive sweeteners Macronutrients Micronutrients Meal planning Snack planning |
| 23 | BC Renal Agency | 2016 | Diabetes Kidney-Friendly Shopping List | P/T gov. | Handout | None | No | Blood sugar management Nephropathy | Carbohydrate counting Label reading Non-nutritive sweeteners Macronutrients Micronutrients |
| 24 | BETTER Project | 2022 | Treatment of hypoglycemia | National NGO | Handout | French | No | Blood sugar management | Carbohydrate counting Macronutrients Snack planning |
| 25 | BETTER Project | 2022 | Examples of Snacks in Various Situations | National NGO | Handout | French | No | Blood sugar management Gastrointestinal health | Macronutrients Snack planning |
| 26 | Calgary Health Region | 2008 | Healthy Eating with Type 2 Diabetes | P/T gov. | Handout | None | No | Blood sugar management Cardiovascular disease Weight management | Canada's Food Guide Balanced plate method Glycemic index Carbohydrate counting Label reading Non-nutritive sweeteners Macronutrients Meal planning Snack planning |
| 27 | Canadian Diabetes Association | 2008 | South Asian Food Pictures | National NGO | Handout | Hindi; Punjabi; Tamil; Urdu | South & Southeast Asian | Blood sugar management | Carbohydrate counting Macronutrients |
| 28 | Canadian Diabetes Association | 2013 | Just the Basics: Aboriginal Version | National NGO | Handout | French; Innuinaqtun; Inuktitut; Ojibwe; Plains Cree | Indigenous | Blood sugar management Cardiovascular disease Weight management Mental health | Balanced plate method Non-nutritive sweeteners Macronutrients Meal planning |
| 29 | Canadian Diabetes Association and Government of Ontario | 2013 | Managing Your Cholesterol | National NGO | Handout | French; Hindi; Oji-Cree; Ojibwe; Plains Cree; Polish; Portuguese; Chinese (Simplified); Chinese (Traditional); Spanish; Tamil; Urdu | No | Blood sugar management Cardiovascular disease Weight management Hypertension | Macronutrients General healthy eating |
| 30 | Canadian Diabetes Association and Government of Ontario | 2013 | Healthy Eating: The Basics | National NGO | Handout | French; Hindi; Oji-Cree; Ojibwe; Plains Cree; Polish; Portuguese; Chinese (Simplified); Chinese (Traditional); Spanish; Tamil; Urdu | No | Blood sugar management Cardiovascular disease Weight management | Canada's Food Guide Balanced plate method Label reading Eating away from home Non-nutritive sweeteners Macronutrients Meal planning Snack planning |
| 31 | Canadian Diabetes Association, Government of Ontario | 2013 | Maintaining a Healthy Weight | National NGO | Handout | French; Hindi; Oji-Cree; Ojibwe; Plains Cree; Polish; Portuguese; Chinese (Simplified); Chinese (Traditional); Spanish; Tamil; Urdu | No | Blood sugar management Cardiovascular disease Weight management Hypertension Neuropathy Retinopathy | Popular weight loss diets Eating away from home Macronutrients Meal planning Snack planning |
| 32 | Cree Board of Health and Social Services of James Bay | 2018 | Puzzle Out Your Carbs | P/T gov. | Handout | None | Indigenous | Blood sugar management | Balanced plate method Carbohydrate counting Label reading |
| 33 | Cree Board of Health and Social Services of James Bay | 2017 | Eat Healthy! Live Healthy! The Plate Method | P/T gov. | Handout | None | Indigenous | Blood sugar management | Balanced plate method |
| 34 | Cree Board of Health and Social Services of James Bay | nd | What is Diabetes? A Guide to Understanding Type 2 Diabetes | P/T gov. | Handout | None | Indigenous | Blood sugar management Weight management | Balanced plate method  Macronutrients |
| 35 | Diabetes Canada | 2012 | Beyond the Basics: Meal Planning for Healthy Eating and Diabetes Management | National NGO | Handout | French | No | Blood sugar management Weight management | Balanced plate method Carbohydrate counting Label reading Non-nutritive sweeteners Macronutrients Meal planning |
| 36 | Diabetes Canada | 2018 | Sugars and Sweeteners | National NGO | Handout | French; Chinese (Traditional) | No | Blood sugar management | Macronutrients |
| 37 | Diabetes Canada | nd | Stay Safe When You Have Diabetes and Are Sick or at Risk of Dehydration | National NGO | Handout | French | No | Blood sugar management Gastrointestinal health | Label reading Non-nutritive sweeteners Macronutrients |
| 38 | Diabetes Canada | 2018 | Hypoglycemia: low blood sugar in adults | National NGO | Handout | French | No | Blood sugar management | Macronutrients Snack planning |
| 39 | Diabetes Canada | nd | Drive Safe with Diabetes | National NGO | Handout | French | No | Blood sugar management | Macronutrients Snack planning |
| 40 | Diabetes Canada | 2018 | Managing Weight and Diabetes | National NGO | Handout | French; Chinese (Traditional) | No | Blood sugar management Weight management Cardiovascular disease Hypertension Neuropathy Retinopathy Mental health | Canada's Food Guide Popular weight loss diets Balanced plate method Eating away from home Macronutrients Meal planning Snack planning |
| 41 | Diabetes Canada | 2018 | High blood pressure and diabetes | National NGO | Handout | Chinese (Traditional) | No | Blood sugar management Cardiovascular disease Weight management Hypertension Mental health | DASH diet Label reading Macronutrients Micronutrients Meal planning |
| 42 | Diabetes Canada | 2018 | Cholesterol and diabetes | National NGO | Handout | French; Chinese (Simplified); Chinese (Traditional) | No | Blood sugar management Weight management Cardiovascular disease | Glycemic index Label reading Macronutrients General healthy eating |
| 43 | Diabetes Canada | 2018 | Basic Carbohydrate Counting for Diabetes Management | National NGO | Handout | French; Chinese (Traditional) | No | Blood sugar management Cardiovascular disease Weight management | Carbohydrate counting Label reading Macronutrients |
| 44 | Diabetes Canada | 2022 | Healthy Eating with Diabetes: South Asian | National NGO | Handout | Hindi; Punjabi; Tamil; Urdu | South & Southeast Asian | Blood sugar management Cardiovascular disease Weight management Mental health | Balanced plate method Eating away from home Non-nutritive sweeteners Macronutrients Meal planning Snack planning |
| 45 | Diabetes Canada | 2022 | Healthy Eating with Diabetes: Chinese | National NGO | Handout | Chinese (Simplified); Chinese (Traditional) | East Asian | Blood sugar management | Balanced plate method Eating away from home Non-nutritive sweeteners Macronutrients Meal planning Snack planning |
| 46 | Diabetes Canada | 2022 | Healthy Eating with Diabetes: African and Caribbean | National NGO | Handout | French | African, Caribbean | Blood sugar management | Balanced plate method Eating away from home Non-nutritive sweeteners Macronutrients Meal planning Snack planning |
| 47 | Diabetes Canada | 2021 | The Balanced Food Plate | National NGO | Handout | Arabic; French; Hindi; Italian; Portuguese; Punjabi; Chinese (Simplified); Chinese (Traditional); Spanish; Tamil; Urdu | No | Blood sugar management | Balanced plate method Meal planning Snack planning |
| 48 | Diabetes Canada | 2018 | Just the basics | National NGO | Handout | French; Chinese (Traditional) | No | Blood sugar management Cardiovascular disease Weight management | Mediterranean diet Canada's Food Guide DASH diet Balanced plate method Glycemic index Carbohydrate counting Non-nutritive sweeteners Macronutrients Meal planning Snack planning |
| 49 | Diabetes Canada | 2018 | Just the basics: South Asian | National NGO | Handout | None | South & Southeast Asian | Blood sugar management | Mediterranean diet DASH diet Nordic diet Vegetarian diet Balanced plate method Carbohydrate counting Meal planning Snack planning |
| 50 | Diabetes Canada | 2018 | Just the basics: Latin American | National NGO | Handout | Portuguese; None | Latin American | Blood sugar management | Mediterranean diet DASH diet Nordic diet Vegetarian diet Balanced plate method Carbohydrate counting Glycemic index Snack planning |
| 51 | Diabetes Canada | 2018 | Eating Away From Home | National NGO | Handout | French | No | Blood sugar management Cardiovascular disease Hypertension | Canada's Food Guide Balanced plate method Label reading Eating away from home Macronutrients Micronutrients Meal planning Snack planning |
| 52 | Diabetes Canada | 2022 | Diabetes and Dining Out: African and Caribbean | National NGO | Handout | French | African, Caribbean | Blood sugar management | Canada's Food Guide Carbohydrate counting Eating away from home Macronutrients Micronutrients Meal planning Snack planning |
| 53 | Diabetes Canada | 2022 | Diabetes and Dining Out: Chinese | National NGO | Handout | Chinese (Simplified); Chinese (Traditional) | East Asian | Blood sugar management | Canada's Food Guide Carbohydrate counting Eating away from home Macronutrients Micronutrients Meal planning Snack planning |
| 54 | Diabetes Canada | 2022 | Diabetes and Dining Out: South Asian | National NGO | Handout | Hindi; Punjabi; Tamil; Urdu | South & Southeast Asian | Blood sugar management | Canada's Food Guide Carbohydrate counting Eating away from home Macronutrients Micronutrients Meal planning Snack planning |
| 55 | Diabetes Canada | 2018 | Glycemic Index Food Guide | National NGO | Handout | French; Chinese (Traditional) | No | Blood sugar management Cardiovascular disease Weight management | Balanced plate method Glycemic index Macronutrients Meal planning |
| 56 | Diabetes Canada | 2014 | Handy Portion Guide | National NGO | Handout | None | No | Blood sugar management | Meal planning |
| 57 | Diabetes Canada | 2018 | Webinar: Curb Your Cravings | National NGO | Audiovisual | None | No | Blood sugar management Cardiovascular disease Weight management Mental health | Canada's Food Guide Balanced plate method Label reading Eating away from home Macronutrients Meal planning Snack planning |
| 58 | Diabetes Canada | 2016 | What is the Glycemic Index? | National NGO | Audiovisual | None | No | Blood sugar management Weight management Cardiovascular disease | Glycemic index Non-nutritive sweeteners Macronutrients Micronutrients Meal planning Snack planning |
| 59 | Diabetes Canada | 2015 | The Mediterranean Diet: Is It Right For You? | National NGO | Audiovisual | None | No | Blood sugar management Weight management Cardiovascular disease Hypertension | Mediterranean diet Canada's Food Guide Vegetarian diet Glycemic index Macronutrients Micronutrients Meal planning Snack planning Gluten-free diet |
| 60 | Diabetes Canada | 2016 | Pulses: A New Superfood | National NGO | Audiovisual | None | No | Blood sugar management Cardiovascular disease Weight management Gastrointestinal health | Vegetarian diet Glycemic index Carbohydrate counting Macronutrients Micronutrients Meal planning Snack planning |
| 61 | Diabetes Canada | 2017 | Making the Most of Your Food Dollar | National NGO | Audiovisual | None | No | Blood sugar management Cardiovascular disease | Canada's Food Guide Balanced plate method Label reading Eating away from home Macronutrients Meal planning Snack planning |
| 62 | Diabetes Canada | 2017 | Living Well With Diabetes From a South Asian Perspective | National NGO | Audiovisual | None | South & Southeast Asian | Blood sugar management Weight management Cardiovascular disease Gastrointestinal health Hypertension Nephropathy Neuropathy Retinopathy Mental health | Canada's Food Guide Vegetarian diet Label reading Macronutrients Micronutrients Meal planning |
| 63 | Diabetes Canada | 2017 | Living Well With Diabetes From a Chinese Perspective | National NGO | Audiovisual | None | East Asian | Blood sugar management Weight management Cardiovascular disease Gastrointestinal health | Non-nutritive sweeteners Macronutrients Eating away from hoime Meal planning |
| 64 | Diabetes Canada | 2017 | Living Well With Diabetes From an Aboriginal Perspective | National NGO | Audiovisual | None | Indigenous | Blood sugar management Cardiovascular disease Weight management Hypertension Nephropathy Mental health | Canada's Food Guide Balanced plate method Label reading Eating away from home Macronutrients Micronutrients Meal planning Snack planning |
| 65 | Diabetes Canada | 2017 | Eat Smart to Manage Blood Glucose | National NGO | Audiovisual | None | No | Blood sugar management Cardiovascular disease Weight management Gastrointestinal health Nephropathy Neuropathy Retinopathy | Vegetarian diet Balanced plate method Carbohydrate counting Label reading Eating away from home Non-nutritive sweeteners Macronutrients Micronutrients Meal planning Snack planning |
| 66 | Diabetes Canada | 2018 | Healthy Living Webinar: African Caribbean Perspective | National NGO | Audiovisual | None | African, Caribbean | Blood sugar management Cardiovascular disease Weight management Gastrointestinal health Hypertension Mental health | Mediterranean diet Canada's Food Guide DASH diet Portfolio diet Vegetarian diet Balanced plate method Glycemic index Label reading Eating away from home Macronutrients Micronutrients Meal planning Snack planning |
| 67 | Diabetes Canada | 2018 | Healthy Living Webinar: South Asian Perspective | National NGO | Audiovisual | None | South & Southeast Asian | Blood sugar management Cardiovascular disease Weight management Hypertension Mental health | Canada's Food Guide Balanced plate method Glycemic index Label reading Macronutrients Micronutrients Meal planning Snack planning |
| 68 | Diabetes Canada | 2022 | Intermittent Fasting: Ask the Experts from Diabetes Canada | National NGO | Audiovisual | None | No | Blood sugar management Weight management | Intermittent fasting |
| 69 | Diabetes Canada | 2022 | Low-Carb & Keto Diets: Ask the Experts from Diabetes Canada | National NGO | Audiovisual | None | No | Blood sugar management Cardiovascular disease Weight management | Low carbohydrate diet Macronutrients Micronutrients |
| 70 | Diabetes Canada | 2021 | Gastroparesis: Ask the Experts from Diabetes Canada | National NGO | Audiovisual | None | No | Blood sugar management Gastroparesis Neuropathy | Macronutrients Meal planning |
| 71 | Diabetes Canada | 2018 | Webinar: Carb Counting Made Easy | National NGO | Audiovisual | None | No | Blood sugar management | Balanced plate method Glycemic index Carbohydrate counting Label reading Eating away from home Macronutrients |
| 72 | Diabetes Canada | 2021 | 7-Day Caribbean Meal Plan | National NGO | Handout | None | Caribbean | Blood sugar management | Macronutrients Meal planning Snack planning |
| 73 | Diabetes Canada | 2021 | 7-day Chinese healthy meal plan | National NGO | Handout | Chinese (Traditional) | East Asian | Blood sugar management | Glycemic index Macronutrients Micronutrients Snack planning |
| 74 | Diabetes Canada | 2021 | 7-Day Low-Carbohydrate Meal Plan | National NGO | Handout | None | No | Blood sugar management Weight management | Low carbohydrate diet Glycemic index Macronutrients Meal planning Snack planning |
| 75 | Diabetes Canada | 2021 | 7-Day Dairy-Free Meal Plan | National NGO | Handout | None | No | Blood sugar management | Glycemic index Macronutrients Micronutrients Meal planning Snack planning |
| 76 | Diabetes Canada | 2021 | 7-Day Gluten-Free Meal Plan | National NGO | Handout | None | No | Blood sugar management | Gluten-free diet Macronutrients Meal planning Snack planning |
| 77 | Diabetes Canada | 2021 | 7-Day Mediterranean Meal Plan | National NGO | Handout | None | No | Blood sugar management | Mediterranean diet Glycemic index Macronutrients Meal planning Snack planning |
| 78 | Diabetes Canada | 2021 | 7-Day South Asian Meal Plan | National NGO | Handout | None | South & Southeast Asian | Blood sugar management | Macronutrients Meal planning Snack planning |
| 79 | Diabetes Canada | 2021 | 7-Day Vegetarian Meal Plan | National NGO | Handout | None | No | Blood sugar management | Vegetarian diet Glycemic index Meal planning Snack planning |
| 80 | Diabetes Canada | 2021 | 7-Day Vegan Meal Plan | National NGO | Handout | None | No | Blood sugar management | Vegetarian diet Glycemic index Meal planning Snack planning |
| 81 | Diabetes Canada | 2021 | 7-Day Healthy Meal Plan | National NGO | Handout | None | No | Blood sugar management | Glycemic index Macronutrients Meal planning Snack planning |
| 82 | Diabetes Canada | nd | Basic Meal Planning | National NGO | Web article | None | No | Blood sugar management Weight management Cardiovascular disease Hypertension | Mediterranean diet Canada's Food Guide DASH diet Glycemic index Carbohydrate counting Non-nutritive sweeteners Macronutrients Micronutrients Meal planning Snack planning |
| 83 | Diabetes Canada | nd | Dining Out | National NGO | Web article | None | No | Blood sugar management | Canada's Food Guide Balanced plate method Eating away from home Macronutrients Meal planning Snack planning |
| 84 | Diabetes Canada | nd | Healthy Eating | National NGO | Web article | None | No | Blood sugar management Cardiovascular disease Weight management | Canada's Food Guide Balanced plate method Eating away from home Non-nutritive sweeteners Macronutrients Meal planning Snack planning |
| 85 | Diabetes Canada | nd | The glycemic index (GI) | National NGO | Web article | None | No | Blood sugar management Cardiovascular disease Weight management | Glycemic index Macronutrients Meal planning |
| 86 | University Health Network (Diabetes College) | nd | Food Labels | P/T gov. | Web article | French | No | Blood sugar management Cardiovascular disease Hypertension | Label reading Macronutrients Micronutrients |
| 87 | University Health Network (Diabetes College) | nd | Eat Less Salt | P/T gov. | Web article | French | No | Blood sugar management Hypertension | DASH diet Label reading Eating away from home Micronutrients Meal planning |
| 88 | University Health Network (Diabetes College) | nd | Eat More Fibre | P/T gov. | Web article | French | No | Blood sugar management Cardiovascular disease Gastrointestinal health Hypertension | Glycemic index Macronutrients Meal planning Snack planning |
| 89 | University Health Network (Diabetes College) | nd | Healthy Snacks List | P/T gov. | Web article | French | No | Blood sugar management | Snack planning |
| 90 | University Health Network (Diabetes College) | nd | Manage Cholesterol | P/T gov. | Web article | French; None | No | Blood sugar management Cardiovascular disease Hypertension | Mediterranean diet Macronutrients Macronutrients |
| 91 | University Health Network (Diabetes College) | nd | Mindful and Intuitive Eating | P/T gov. | Web article | French | No | Blood sugar management Mental health | Meal planning |
| 92 | Diabetes Québec | 2022 | Low Blood Sugar: Symptoms and Actions to Take | P/T NGO | Handout | French | No | Blood sugar management | Macronutrients Macronutrients Snack planning |
| 93 | Diabetes Québec | 2018 | Adapting Your Recipes | P/T NGO | Web article | French | No | Blood sugar management Cardiovascular disease Weight management | Balanced plate method Macronutrients Meal planning |
| 94 | Diabetes Québec | 2018 | Diabetes at the Supermarket | P/T NGO | Web article | French | No | Blood sugar management | Balanced plate method Label reading Meal planning Snack planning |
| 95 | Diabetes Québec | 2018 | Food Labels 101 | P/T NGO | Web article | French | No | Blood sugar management Cardiovascular disease | Label reading Macronutrients Micronutrients |
| 96 | Diabetes Québec | 2018 | Alcoholic Coffees | P/T NGO | Web article | French | No | Blood sugar management | Macronutrients General healthy eating |
| 97 | Diabetes Québec | 2018 | Carbohydrates | P/T NGO | Web article | French | No | Blood sugar management | Carbohydrate counting Label reading Eating away from home Macronutrients Micronutrients Meal planning |
| 98 | Diabetes Québec | 2018 | Coconut Oil | P/T NGO | Web article | French | No | Blood sugar management Cardiovascular disease | Macronutrients General healthy eating |
| 99 | Diabetes Québec | 2018 | Fats | P/T NGO | Web article | French | No | Blood sugar management Cardiovascular disease Weight management | Label reading Macronutrients Micronutrients General healthy eating |
| 100 | Diabetes Québec | 2018 | Dietary Fibre | P/T NGO | Web article | French | No | Blood sugar management Weight management Cardiovascular disease Gastrointestinal health | Label reading Macronutrients Meal planning Snack planning |
| 101 | Diabetes Québec | 2018 | Flax, Chia and Hemp Seeds | P/T NGO | Web article | French | No | Blood sugar management Cardiovascular disease Weight management | Macronutrients General healthy eating |
| 102 | Diabetes Québec | 2018 | Fruit | P/T NGO | Web article | French | No | Blood sugar management Weight management | Label reading Macronutrients Micronutrients General healthy eating |
| 103 | Diabetes Québec | 2018 | Glycemic Index | P/T NGO | Web article | French | No | Blood sugar management Cardiovascular disease | Glycemic index Carbohydrate counting Macronutrients |
| 104 | Diabetes Québec | 2018 | Greek Yogurt | P/T NGO | Web article | French | No | Blood sugar management Cardiovascular disease | Label reading Macronutrients Micronutrients Snack planning |
| 105 | Diabetes Québec | 2018 | Hot Drinks | P/T NGO | Web article | French | No | Blood sugar management | Carbohydrate counting Label reading Eating away from home Macronutrients |
| 106 | Diabetes Québec | 2018 | How to Choose a Whole-wheat Bread | P/T NGO | Web article | French | No | Blood sugar management Gastrointestinal health | Label reading Macronutrients Micronutrients General healthy eating |
| 107 | Diabetes Québec | 2018 | How to Choose the Best Breakfast Cereals | P/T NGO | Web article | French | No | Blood sugar management Cardiovascular disease | Label reading Macronutrients Micronutrients General healthy eating |
| 108 | Diabetes Québec | 2018 | Legumes and Pulses | P/T NGO | Web article | French | No | Blood sugar management Cardiovascular disease Weight management Gastrointestinal health Hypertension | Macronutrients Micronutrients General healthy eating |
| 109 | Diabetes Québec | 2018 | Maple Syrup: not a Miracle Food After All | P/T NGO | Web article | French | No | Blood sugar management | Glycemic index Macronutrients Micronutrients General healthy eating |
| 110 | Diabetes Québec | 2018 | Meat and its Alternatives | P/T NGO | Web article | French | No | Blood sugar management Cardiovascular disease Nephropathy | Label reading Macronutrients Micronutrients General healthy eating |
| 111 | Diabetes Québec | 2018 | Milk and its Alternatives | P/T NGO | Web article | French | No | Blood sugar management | Macronutrients Micronutrients General healthy eating |
| 112 | Diabetes Québec | 2018 | Omega-3 Fats | P/T NGO | Web article | French | No | Blood sugar management Cardiovascular disease Hypertension | Macronutrients Micronutrients Meal planning |
| 113 | Diabetes Québec | 2018 | Sodium | P/T NGO | Web article | French | No | Blood sugar management Weight management Cardiovascular disease Hypertension | Label reading Eating away from home Micronutrients General healthy eating |
| 114 | Diabetes Québec | 2018 | Spices and Culinary Herbs | P/T NGO | Web article | French | No | Blood sugar management Hypertension | Micronutrients General healthy eating |
| 115 | Diabetes Québec | 2018 | Starches | P/T NGO | Web article | French | No | Blood sugar management | Label reading Macronutrients Meal planning |
| 116 | Diabetes Québec | 2018 | Sugar Substitutes | P/T NGO | Web article | French | No | Blood sugar management Gastrointestinal health | Non-nutritive sweeteners |
| 117 | Diabetes Québec | 2018 | The Composition of Foods | P/T NGO | Web article | French | No | Blood sugar management Weight management Cardiovascular disease Gastrointestinal health | Macronutrients Micronutrients |
| 118 | Diabetes Québec | 2018 | Vegetables | P/T NGO | Web article | French | No | Blood sugar management | Carbohydrate counting Macronutrients Micronutrients Meal planning Snack planning |
| 119 | Diabetes Québec | 2020 | A Practical Guide to Diabetes Management | P/T NGO | Handout | French | No | Blood sugar management Weight management Cardiovascular disease Neuropathy Mental health | Balanced plate method Label reading Macronutrients Meal planning Snack planning |
| 120 | Diabetes Québec | 2018 | Your Pocket Guide to Dining Out | P/T NGO | Report | French | No | Blood sugar management | Balanced plate method Carbohydrate counting Eating away from home Non-nutritive sweeteners Macronutrients Meal planning |
| 121 | Diabetes Québec | 2018 | Snacks | P/T NGO | Web article | French | No | Blood sugar management Mental Health | Snack planning Macronutrients |
| 122 | Diabetes Québec | 2018 | The Balanced Plate | P/T NGO | Web article | French | No | Blood sugar management | Balanced plate method Macronutrients Micronutrients Meal planning Snack planning |
| 123 | Diabetes Québec | 2018 | The DASH Diet | P/T NGO | Web article | French | No | Blood sugar management Cardiovascular disease Weight management Hypertension | Canada's Food Guide DASH diet Balanced plate method Label reading Macronutrients Micronutrients Meal planning Snack planning |
| 124 | Diabetes Québec | 2018 | The Mediterranean Diet | P/T NGO | Web article | French | No | Blood sugar management Cardiovascular disease Hypertension Retinopathy | Mediterranean diet Macronutrients Micronutrients Meal planning |
| 125 | Diabetes Québec | 2018 | Vegetarianism and Diabetes | P/T NGO | Web article | French | No | Blood sugar management Cardiovascular disease Weight management Hypertension | Vegetarian diet Balanced plate method Macronutrients Micronutrients Meal planning |
| 126 | Diabetes Québec | 2022 | Snacks and Diabetes | P/T NGO | Handout | French | No | Blood sugar management Weight management Mental health | Non-nutritive sweeteners Macronutrients Snack planning |
| 127 | Dietitians of Canada - PEN | 2017 | Diabetes: Balancing Meals with Carbohydrate Counting | National NGO | Handout | French | No | Blood sugar management | Carbohydrate counting Macronutrients Meal planning Snack planning |
| 128 | Dietitians of Canada - PEN | 2019 | Eating Guidelines for People with High Blood Cholesterol Levels | National NGO | Handout | French | No | Blood sugar management Cardiovascular disease Weight management | Mediterranean diet Portfolio diet Label reading Macronutrients Meal planning Snack planning |
| 129 | Dietitians of Canada - PEN | 2020 | Using the DASH Diet to Help Lower Blood Pressure | National NGO | Handout | French | No | Blood sugar management Cardiovascular disease Weight management Hypertension Mental health | DASH diet Label reading Macronutrients Micronutrients |
| 130 | Dietitians of Canada - PEN | 2020 | The Mediterranean Diet: A Guide to Healthy Eating | National NGO | Handout | French | No | Blood sugar management Cardiovascular disease | Mediterranean diet Macronutrients Micronutrients Meal planning Snack planning |
| 131 | Dietitians of Canada - PEN | 2016 | Healthy Snack Ideas: Type 2 Diabetes | National NGO | Handout | French | No | Blood sugar management Cardiovascular disease Gastrointestinal health Nephropathy | Canada's Food Guide Label reading Macronutrients Snack planning |
| 132 | Dietitians of Canada - PEN | 2017 | Healthy Snack Ideas: Type 2 Diabetes - Adapted for South Asian Communities | National NGO | Handout | None | South & Southeast Asian | Blood sugar management | Canada's Food Guide Label reading Macronutrients Snack planning |
| 133 | Dietitians of Canada - PEN | 2017 | Healthy Snack Ideas: Type 2 Diabetes - Adapted for Latin American Communities | National NGO | Handout | None | Latin American | Blood sugar management | Canada's Food Guide Label reading Macronutrients Snack planning |
| 134 | Dietitians of Canada - PEN | 2017 | Healthy Snack Ideas: Type 2 Diabetes: Adapted for Chinese Communities | National NGO | Handout | None | East Asian | Blood sugar management | Canada's Food Guide Label reading Macronutrients Snack planning |
| 135 | Dietitians of Canada - PEN | 2017 | Healthy Snack Ideas: Type 2 Diabetes - Adapted for African and Caribbean Communities | National NGO | Handout | None | African, Caribbean | Blood sugar management | Canada's Food Guide Label reading Macronutrients Snack planning |
| 136 | Dietitians of Canada - PEN | 2015 | Healthy Eating in the Workplace for People with Type 2 Diabetes - Adapted for African and Carribean Communities | National NGO | Handout | None | African, Caribbean | Blood sugar management Cardiovascular disease Nephropathy Neuropathy Retinopathy Mental health | Eating away from home Macronutrients Meal planning Snack planning |
| 137 | Dietitians of Canada - PEN | 2015 | Healthy Eating in the Workplace for People with Type 2 Diabetes - Adapted for Latin American Communities | National NGO | Handout | None | Latin American | Blood sugar management | Eating away from home Macronutrients Meal planning Snack planning |
| 138 | Dietitians of Canada - PEN | 2015 | Healthy Eating in the Workplace for People with Type 2 Diabetes - Adapted for Chinese Communities | National NGO | Handout | None | East Asian | Blood sugar management | Eating away from home Macronutrients Meal planning Snack planning |
| 139 | Dietitians of Canada - PEN | 2015 | Healthy Eating in the Workplace for People with Type 2 Diabetes - Adapted for North and South Indian Communities | National NGO | Handout | None | South & Southeast Asian | Blood sugar management | Eating away from home Macronutrients Meal planning Snack planning |
| 140 | Dietitians of Canada - PEN | 2012 | Healthy Eating for Diabetes: Adapted for South Indian Diets | National NGO | Handout | None | South & Southeast Asian | Blood sugar management Cardiovascular disease Weight management Nephropathy Neuropathy Retinopathy | Balanced plate method Glycemic index Label reading Macronutrients Meal planning Snack planning |
| 141 | Dietitians of Canada - PEN | 2012 | Healthy Eating for Diabetes: Adapted for Pakistani Diets | National NGO | Handout | None | Middle Eastern | Blood sugar management | Balanced plate method Glycemic index Label reading Macronutrients Meal planning Snack planning |
| 142 | Dietitians of Canada - PEN | 2012 | Healthy Eating for Diabetes: Adapted for North Indian Diets | National NGO | Handout | None | South & Southeast Asian | Blood sugar management | Balanced plate method Glycemic index Label reading Macronutrients Meal planning Snack planning |
| 143 | Dietitians of Canada - PEN | 2012 | Healthy Eating for Diabetes: Adapted for Latin/Hispanic Community | National NGO | Handout | None | Latin American | Blood sugar management | Balanced plate method Glycemic index Label reading Macronutrients Meal planning Snack planning |
| 144 | Dietitians of Canada - PEN | 2012 | Healthy Eating for Diabetes: Adapted for Gujarati Diets | National NGO | Handout | None | South & Southeast Asian | Blood sugar management | Balanced plate method Glycemic index Label reading Macronutrients Meal planning Snack planning |
| 145 | Dietitians of Canada - PEN | 2012 | Healthy Eating for Diabetes: Adapted for African and Caribbean Diets | National NGO | Handout | None | African, Caribbean | Blood sugar management | Balanced plate method Glycemic index Label reading Macronutrients Meal planning Snack planning |
| 146 | Dietitians of Canada - Unlock | 2019 | What You Need to Know About Sugar | National NGO | Web article | French | No | Blood sugar management | Canada's Food Guide Label reading Macronutrients General healthy eating |
| 147 | Dietitians of Canada - Unlock | 2022 | What Are the Different Types of Sweeteners? Are They Safe? | National NGO | Web article | French | No | Blood sugar management Gastrointestinal health | Carbohydrate counting Label reading Non-nutritive sweeteners Macronutrients General healthy eating |
| 148 | Dietitians of Canada - Unlock | 2018 | What are Ancient Grains and Are They Healthy? | National NGO | Web article | French | No | Blood sugar management Cardiovascular disease Hypertension | Label reading Macronutrients General healthy eating Gluten-free diet |
| 149 | Dietitians of Canada - Unlock | 2022 | Understanding Eggs and Cholesterol | National NGO | Web article | French | No | Blood sugar management Cardiovascular disease | Macronutrients Micronutrients General healthy eating |
| 150 | Dietitians of Canada - Unlock | 2018 | My Menu Planner: Type 2 Diabetes Recipes | National NGO | Handout | French | No | Blood sugar management | Macronutrients Meal planning Snack planning |
| 151 | Dietitians of Canada - Unlock | 2018 | My Menu Planner: Type 2 Diabetes Menu Plan - Male | National NGO | Handout | French | No | Blood sugar management | Carbohydrate counting Macronutrients Meal planning Snack planning |
| 152 | Dietitians of Canada - Unlock | 2018 | My Menu Planner: Type 2 Diabetes Menu Plan - Female | National NGO | Handout | French | No | Blood sugar management | Carbohydrate counting Macronutrients Meal planning Snack planning |
| 153 | Dietitians of Canada - Unlock | 2019 | Tips for healthy snacking for people with diabetes | National NGO | Web article | French | No | Blood sugar management Gastrointestinal health | Canada's Food Guide Non-nutritive sweeteners Macronutrients Micronutrients Snack planning |
| 154 | Dietitians of Canada - Unlock | 2018 | Recipe Makeover: Reducing Sugar in the Kitchen | National NGO | Web article | French | No | Blood sugar management | Canada's Food Guide Non-nutritive sweeteners Snack planning |
| 155 | Dietitians of Canada - Unlock | 2019 | Healthy snack ideas for people with type 2 diabetes -- Small Snacks | National NGO | Web article | French | No | Blood sugar management | Macronutrients Snack planning Non-nutritive sweeteners |
| 156 | Dietitians of Canada - Unlock | 2020 | Healthy snack ideas for people with type 2 diabetes -- Large Snacks | National NGO | Web article | French | No | Blood sugar management | Macronutrients Snack planning Non-nutritive sweeteners |
| 157 | Dietitians of Canada - Unlock | 2019 | Fill Up On Fibre: Meal and Snack Ideas | National NGO | Web article | French | No | Blood sugar management | Canada's Food Guide Macronutrients Meal planning |
| 158 | Dietitians of Canada - Unlock | 2021 | Everything You Need to Know About the Glycemic Index and Choosing Low Glycemic Foods | National NGO | Web article | French | No | Blood sugar management Cardiovascular disease Gastrointestinal health | Macronutrients Meal planning Low glycemic index diet |
| 159 | Dietitians of Canada - Unlock | 2019 | Eating well with Diabetes: South Indian and Sri Lankan Diets | National NGO | Web article | French | South & Southeast Asian | Blood sugar management Cardiovascular disease | Macronutrients Meal planning Snack planning |
| 160 | Dietitians of Canada - Unlock | 2023 | Eating well with Diabetes: North India and Pakistan diets | National NGO | Web article | French | South & Southeast Asian | Blood sugar management | Macronutrients Meal planning Snack planning |
| 161 | Dietitians of Canada - Unlock | 2023 | Eating well with Diabetes: East Asian diets | National NGO | Web article | French | East Asian | Blood sugar management Hypertension | Macronutrients Meal planning Snack planning |
| 162 | Dietitians of Canada - Unlock | 2023 | Eating well with Diabetes: Caribbean and African diets | National NGO | Web article | French | African, Caribbean | Blood sugar management | Macronutrients Meal planning Snack planning |
| 163 | Dietitians of Canada - Unlock | 2018 | Diabetes, Sugar and Sweet Foods | National NGO | Web article | French | No | Blood sugar management | Label reading Macronutrients Micronutrients General healthy eating |
| 164 | Dietitians of Canada - Unlock | 2018 | Diabetes and the Health Benefits of Fibre | National NGO | Web article | French | No | Blood sugar management Cardiovascular disease Weight management Gastrointestinal health | Label reading Macronutrients Meal planning Snack planning |
| 165 | Dietitians of Canada - Unlock | 2018 | Diabetes and Shift Work | National NGO | Web article | French | No | Blood sugar management Cardiovascular disease Weight management Gastrointestinal health Nephropathy Neuropathy Retinopathy Mental health | Glycemic index Eating away from home Macronutrients Meal planning Snack planning |
| 166 | Dietitians of Canada - Unlock | 2018 | Diabetes and Dining Out | National NGO | Web article | French | No | Blood sugar management Weight management | Balanced plate method Label reading Eating away from home Macronutrients Meal planning |
| 167 | Dietitians of Canada - Unlock | 2023 | Diabetes and Carbohydrates | National NGO | Web article | French | No | Blood sugar management Cardiovascular disease Gastrointestinal health Hypertension Nephropathy Retinopathy | Glycemic index Label reading Macronutrients General healthy eating |
| 168 | EatRight Ontario, George Brown College | 2012 | Diverse Foods and Flavours: Healthy Diabetes Recipes from South Asia | P/T NGO | Handout | None | South & Southeast Asian | Blood sugar management Cardiovascular disease Weight management Gastrointestinal health Hypertension Nephropathy Neuropathy Retinopathy | Balanced plate method Macronutrients Micronutrients Meal planning |
| 169 | EatRight Ontario, George Brown College | 2012 | Diverse Foods and Flavours: Healthy Diabetes Recipes from Latin America | P/T NGO | Handout | None | Latin American | Blood sugar management Cardiovascular disease Hypertension | Balanced plate method Glycemic index Macronutrients Micronutrients Meal planning |
| 170 | EatRight Ontario, George Brown College | 2012 | Diverse Foods and Flavours: Healthy Diabetes Recipes from China | P/T NGO | Handout | None | East Asian | Blood sugar management Cardiovascular disease Weight management Hypertension Nephropathy | Vegetarian diet Balanced plate method Label reading Macronutrients Micronutrients |
| 171 | EatRight Ontario, George Brown College | 2012 | Diverse Foods and Flavours: Healthy Diabetes Recipes from the Caribbean | P/T NGO | Handout | French | Caribbean | Blood sugar management Cardiovascular disease | Glycemic index Macronutrients Micronutrients Meal planning |
| 172 | First Nations Health Authority | 2014 | Healthy Food Guidelines For First Nations Communities | P/T NGO | Report | None | Indigenous | Blood sugar management Weight management Cardiovascular disease Hypertension | Canada's Food Guide Balanced plate method Label reading Eating away from home Non-nutritive sweeteners Macronutrients Micronutrients Meal planning Snack planning |
| 173 | Fraser Health | 2023 | South Asian Meal Plan for Diabetes -- 30 gram Carbohydrate | P/T gov. | Handout | Hindi; Punjabi; Urdu | South & Southeast Asian | Blood sugar management | Vegetarian diet Balanced plate method Glycemic index Carbohydrate counting Macronutrients Meal planning Snack planning |
| 174 | Fraser Health | 2023 | 7 Day Meal Plan for Diabetes -- 30 gram Carbohydrate | P/T gov. | Handout | None | No | Blood sugar management | Macronutrients Meal planning |
| 175 | Fraser Health | 2023 | 7 Day Meal Plan for Diabetes -- 45 gram Carbohydrate | P/T gov. | Handout | None | No | Blood sugar management | Macronutrients Meal planning |
| 176 | Fraser Health | 2023 | Low Cost 7-Day Meal Plan for Diabetes -- 45 gram carbohydrate meals | P/T gov. | Handout | None | No | Blood sugar management | Carbohydrate counting Macronutrients Meal planning |
| 177 | Fraser Health | 2019 | Carbohydrate Choices | P/T gov. | Handout | None | No | Blood sugar management | Carbohydrate counting Macronutrients Meal planning Snack planning |
| 178 | Fraser Health | 2022 | Carbohydrate Counting: Advanced | P/T gov. | Handout | None | No | Blood sugar management | Carbohydrate counting Label reading Non-nutritive sweeteners Macronutrients |
| 179 | Fraser Health | 2018 | Healthy Snack Ideas | P/T gov. | Handout | Punjabi | No | Blood sugar management | Label reading Macronutrients Snack planning |
| 180 | Fraser Health | 2018 | Protein and Diabetes | P/T gov. | Handout | None | No | Blood sugar management Weight management Nephropathy | Macronutrients General healthy eating |
| 181 | Fraser Health, Vancouver Coastal Health, Providence Health Care | 2020 | On the Road to Diabetes Health: An information booklet for people with Type 1 or Type 2 Diabetes | P/T gov. | Handout | Farsi-Persian; Punjabi; Chinese (Simplified) | No | Blood sugar management Cardiovascular disease Weight management Gastrointestinal health Hypertension Mental health | Balanced plate method Glycemic index Carbohydrate counting Label reading Eating away from home Non-nutritive sweeteners Macronutrients Micronutrients Meal planning Snack planning |
| 182 | Government of Northwest Territories | 2017 | Healthy Eating & Healthy Weight Guide | P/T gov. | Report | French | Indigenous | Blood sugar management Weight management Mental health | Canada's Food Guide Balanced plate method Label reading Eating away from home Macronutrients Micronutrients Meal planning Snack planning |
| 183 | Government of Nunavut | 2011 | Nunavut Food Guide | P/T gov. | Handout | French; Innuinaqtun; Inuktitut | Indigenous | Blood sugar management | Canada's Food Guide Macronutrients Meal planning |
| 184 | Government of Nunavut | 2013 | Nutrition Fact Sheet Series: Inuit Traditional Foods | P/T gov. | Handout | None | Indigenous | Blood sugar management Cardiovascular disease Weight management Gastrointestinal health Hypertension Nephropathy | Macronutrients Micronutrients Meal planning Vegetarian diet |
| 185 | Government of Nunavut | nc | Healthy Eating and Diabetes | P/T gov. | Handout | None | Indigenous | Blood sugar management | Meal planning |
| 186 | Diabetes Québec | 2017 | Meal Planning for People with Diabetes | P/T gov. | Handout | French | No | Blood sugar management Cardiovascular disease Weight management Gastrointestinal health Hypertension | Balanced plate method Carbohydrate counting Label reading Non-nutritive sweeteners Macronutrients Micronutrients Meal planning Snack planning |
| 187 | Diabetes Québec | 2014 | Meal Planning for People with Diabetes at a Glance | P/T gov. | Handout | French | No | Blood sugar management Cardiovascular disease Weight management Nephropathy Neuropathy Retinopathy | Balanced plate method Carbohydrate counting Eating away from home Non-nutritive sweeteners Macronutrients Micronutrients Meal planning Snack planning |
| 188 | Diabetes Québec | 2022 | A Closer Look: Diet for People Living with Diabetes | P/T gov. | Handout | French | No | Blood sugar management Cardiovascular disease Hypertension Nephropathy Neuropathy Retinopathy | Balanced plate method Carbohydrate counting Label reading Non-nutritive sweeteners Macronutrients Micronutrients Meal planning Snack planning |
| 189 | Government of Saskatchewan | 2014 | Nutrition Tips for those with Diabetes and Chronic Kidney Disease | P/T gov. | Handout | None | No | Blood sugar management Cardiovascular disease Weight management Hypertension Nephropathy Mental health | Canada's Food Guide Balanced plate method Label reading Macronutrients Micronutrients Meal planning |
| 190 | Government of Yukon | 2015 | Yukon Diabetes Resource Guide: Positive Steps to Manage Your Diabetes | P/T gov. | Report | French | Indigenous | Blood sugar management Cardiovascular disease Weight management | Canada's Food Guide Balanced plate method Non-nutritive sweeteners Macronutrients Snack planning |
| 191 | Hamilton Health Sciences | 2014 | The No Sugar Added Diet | P/T NGO | Handout | None | No | Blood sugar management Weight management | Canada's Food Guide Non-nutritive sweeteners Meal planning Snack planning |
| 192 | Hamilton Health Sciences | 2013 | Portfolio Diet | P/T NGO | Handout | None | No | Blood sugar management Cardiovascular disease Hypertension | Portfolio diet Vegetarian diet Eating away from home  Macronutrients Meal planning |
| 193 | Hamilton Health Sciences | 2017 | Sweeteners and Sugar Substitutes | P/T NGO | Handout | None | No | Blood sugar management | Non-nutritive sweeteners Macronutrients |
| 194 | Hamilton Health Sciences | 2017 | Plant sterols - could they be right for you? | P/T NGO | Handout | None | No | Blood sugar management Cardiovascular disease Weight management | Canada's Food Guide Vegetarian diet Macronutrients General healthy eating |
| 195 | Hamilton Health Sciences | 2008 | Diabetes Care Centre - South Asian Meal Planning Guide | P/T NGO | Handout | None | South & Southeast Asian | Blood sugar management | Carbohydrate counting Macronutrients Meal planning Snack planning |
| 196 | Hamilton Health Sciences | 2012 | Low blood sugar (Hypoglycemia) | P/T NGO | Handout | None | No | Blood sugar management | Macronutrients Snack planning |
| 197 | Hamilton Health Sciences | 2021 | Living well with Diabetes: Information to help you understand and learn to live with diabetes | P/T NGO | Handout | None | No | Blood sugar management Cardiovascular disease Weight management Gastrointestinal health | Balanced plate method Carbohydrate counting Non-nutritive sweeteners Macronutrients Micronutrients Meal planning Snack planning |
| 198 | Hamilton Health Sciences | 2014 | How do I read food labels? | P/T NGO | Handout | None | No | Blood sugar management | Label reading Macronutrients |
| 199 | Hamilton Health Sciences | 2011 | The Glycemic Index of Foods | P/T NGO | Handout | None | No | Blood sugar management Cardiovascular disease Weight management | Glycemic index Macronutrients Micronutrients General healthy eating |
| 200 | Hamilton Health Sciences | 2017 | Blood pressure and your health: The DASH diet and other lifestyle changes | P/T NGO | Handout | None | No | Blood sugar management Gastrointestinal health Hypertension | DASH diet Label reading  Macronutrients Micronutrients Meal planning Snack planning |
| 201 | Hamilton Health Sciences | 2010 | Counting carbohydrates in your favourite recipes | P/T NGO | Handout | None | No | Blood sugar management | Carbohydrate counting Non-nutritive sweeteners Macronutrients |
| 202 | Hamilton Health Sciences | 2008 | Gluten-free Recipe Substitutions: Diabetes and Celiac Disease | P/T NGO | Handout | None | No | Blood sugar management | Label reading Macronutrients Gluten-free diet |
| 203 | Hamilton Health Sciences | 2017 | Carbohydrate Counting | P/T NGO | Handout | Arabic | No | Blood sugar management | Carbohydrate counting Label reading |
| 204 | Hamilton Health Sciences | 2016 | Carbohydrate (Carb) Counting -- The Basics | P/T NGO | Handout | None | No | Blood sugar management Weight management | Balanced plate method Carbohydrate counting |
| 205 | Hamilton Health Sciences | 2013 | Gastroparesis | P/T NGO | Handout | None | No | Blood sugar management Gastrointestinal health Gastroparesis | Label reading Macronutrients Meal planning |
| 206 | Health Canada | 2011 | Using the Nutrition Facts Table: % Daily Value | National gov. | Handout | French | No | Blood sugar management | Label reading Macronutrients Micronutrients |
| 207 | Health Canada | 2007 | Eating Well with Canada's Food Guide: First Nations, Inuit and Métis | National gov. | Handout | French; Inuktitut; Ojibwe; Plains Cree | Indigenous | Blood sugar management Mental health | Canada's Food Guide Macronutrients Micronutrients General healthy eating |
| 208 | Health Canada, Aboriginal Diabetes Initiative | 2008 | Diabetes: What You Eat Matters | National gov. | Handout | French | Indigenous | Blood sugar management | Canada's Food Guide Label reading Macronutrients General healthy eating |
| 209 | HealthLinkBC | 2021 | Diabetes: Coping With Your Feelings About Your Diet | P/T gov. | Web article | None | No | Blood sugar management Mental health | Meal planning |
| 210 | HealthLinkBC | 2021 | Diabetes: Eating Low-Glycemic Foods | P/T gov. | Web article | None | No | Blood sugar management | Glycemic index Balanced plate method Carbohydrate counting Eating away from home Macronutrients Micronutrients Meal planning |
| 211 | HealthLinkBC | 2021 | Eating Out When You Have Diabetes | P/T gov. | Web article | None | No | Blood sugar management | Vegetarian diet Glycemic index Eating away from home Macronutrients Meal planning |
| 212 | HealthLinkBC | 2021 | Quick Tips: Smart Snacking When You Have Diabetes | P/T gov. | Web article | None | No | Blood sugar management | Label reading Macronutrients Snack planning |
| 213 | HealthLinkBC | 2021 | Reading Food Labels When You Have Diabetes | P/T gov. | Web article | None | No | Blood sugar management Cardiovascular disease Hypertension Nephropathy | Carbohydrate counting Label reading Macronutrients Micronutrients |
| 214 | Indigenous Diabetes Health Circle | 2022 | Indigenous Diabetes Health Circle IDHC Recipe Collection | P/T NGO | Handout | None | Indigenous | Blood sugar management | Macronutrients Micronutrients Meal planning Snack planning Eating away from home Gluten-free diet Vegetarian diet |
| 215 | Indigenous Diabetes Health Circle | 2023 | Food is our Medicine - Making it Sacred Cookbook | P/T NGO | Handout | None | Indigenous | Blood sugar management Weight management Cardiovascular disease Gastrointestinal health Mental health | Balanced plate method Label reading Macronutrients Micronutrients Meal planning Gluten-free diet Vegetarian diet |
| 216 | Indigenous Diabetes Health Circle | 2022 | Make it Sacred: Healthy Living Resource & 30-Day Wellness Log | P/T NGO | Handout | None | Indigenous | Blood sugar management Cardiovascular disease Weight management Mental health | Canada's Food Guide Balanced plate method Label reading Macronutrients Micronutrients Meal planning Snack planning |
| 217 | Indigenous Sport, Physical Activity & Recreation Council | 2022 | Healthy Lifestyle Actions for Nutrition & Healthy Eating | National NGO | Handout | None | Indigenous | Blood sugar management Cardiovascular disease Weight management Gastrointestinal health Mental health | Canada's Food Guide Macronutrients Meal planning Snack planning |
| 218 | Indigenous Sport, Physical Activity & Recreation Council | 2022 | Healthy Lifestyle Actions to Manage Diabetes | National NGO | Handout | None | Indigenous | Blood sugar management | Canada's Food Guide Macronutrients Meal planning |
| 219 | Indigenous Sport, Physical Activity & Recreation Council | 2022 | Healthy Lifestyle Actions to Manage Cholesterol | National NGO | Handout | None | Indigenous | Blood sugar management Cardiovascular disease Mental health | Vegetarian diet Macronutrients General healthy eating |
| 220 | Indigenous Sport, Physical Activity & Recreation Council | 2022 | Healthy Lifestyle Actions to Reduce Blood Pressure | National NGO | Handout | None | Indigenous | Blood sugar management Cardiovascular disease Hypertension Nephropathy Mental health | DASH diet Label reading Eating away from home Micronutrients |
| 221 | Island Health | 2018 | Living Well: Tips for nourishing your body, mind and spirit | P/T gov. | Handout | None | Indigenous | Blood sugar management Mental health | Label reading Macronutrients Micronutrients General healthy eating |
| 222 | Island Health | 2018 | Healthy Eating Active Living | P/T gov. | Handout | None | Indigenous | Blood sugar management Weight management | Canada's Food Guide General healthy eating |
| 223 | Island Health | 2016 | Quench Your Thirst | P/T gov. | Handout | None | Indigenous | Blood sugar management | Snack planning General healthy eating |
| 224 | Island Health | 2017 | Setting the Table: Includes a Shopping List, Delicious Meals and Healthy Eating Tips! | P/T gov. | Handout | None | Indigenous | Blood sugar management | Macronutrients Micronutrients Meal planning Snack planning |
| 225 | LMC Diabetes & Endocrinology | 2020 | LMC Guide to Glycemic Index | National NGO | Handout | None | No | Blood sugar management Cardiovascular disease | Balanced plate method Glycemic index Macronutrients |
| 226 | LMC Diabetes & Endocrinology | ? | Eating on a Budget | National NGO | Handout | None | No | Blood sugar management | Macronutrients Micronutrients Meal planning |
| 227 | LMC Diabetes & Endocrinology | 2017 | Guide to Reading Nutrition Labels | National NGO | Handout | None | No | Blood sugar management | Carbohydrate counting Label reading Macronutrients |
| 228 | LMC Diabetes & Endocrinology | 2018 | Top 5 Tips for Dining Out | National NGO | Handout | None | No | Blood sugar management Cardiovascular disease Hypertension | Balanced plate method Label reading Eating away from home Macronutrients Meal planning Snack planning |
| 229 | LMC Diabetes & Endocrinology | 2022 | Carb Counting: Top Tips to Get Started | National NGO | Handout | None | No | Blood sugar management | Glycemic index Carbohydrate counting Label reading Non-nutritive sweeteners Macronutrients |
| 230 | LMC Diabetes & Endocrinology | 2017 | Tips for a Healthy Heart | National NGO | Handout | None | No | Blood sugar management Cardiovascular disease Hypertension Mental health | Label reading Eating away from home Macronutrients Micronutrients General healthy eating |
| 231 | LMC Diabetes & Endocrinology | 2023 | Focus on Fibre | National NGO | Handout | None | No | Blood sugar management Cardiovascular disease Gastrointestinal health | Macronutrients General healty eating |
| 232 | LMC Diabetes & Endocrinology | 2018 | Getting Started with Weight Management: Top 10 Tips | National NGO | Handout | None | No | Blood sugar management Weight management | Macronutrients Meal planning Snack planning |
| 233 | LMC Diabetes & Endocrinology | 2023 | Grocery Shopping Guide | National NGO | Handout | None | No | Blood sugar management | Label reading Macronutrients Micronutrients General healthy eating |
| 234 | LMC Diabetes & Endocrinology | 2022 | Carb Counting Guide - Food List | National NGO | Handout | None | No | Blood sugar management | Carbohydrate counting Macronutrients |
| 235 | LMC Diabetes & Endocrinology | 2017 | LMC Guide to Carbohydrate Sources | National NGO | Handout | None | No | Blood sugar management | Macronutrients |
| 236 | LMC Diabetes & Endocrinology | 2019 | Carb Counting Guide for Fast Food | National NGO | Handout | None | No | Blood sugar management | Carbohydrate counting Macronutrients |
| 237 | LMC Diabetes & Endocrinology | 2019 | Fats & Oils | National NGO | Handout | None | No | Blood sugar management Cardiovascular disease Weight management Hypertension | Label reading Macronutrients Micronutrients General healthy eating |
| 238 | LMC Diabetes & Endocrinology | 2019 | Meal Planning and Ideas | National NGO | Handout | None | No | Blood sugar management | Balanced plate method Macronutrients Meal planning Snack planning |
| 239 | LMC Diabetes & Endocrinology | 2017 | Healthy Home Cooking for Diabetes: Recipe Revisions | National NGO | Handout | None | No | Blood sugar management | Non-nutritive sweeteners Macronutrients Meal planning |
| 240 | LMC Diabetes & Endocrinology | 2022 | LMC Guide to Vegetarian Protein Sources | National NGO | Handout | None | No | Blood sugar management | Vegetarian diet Macronutrients General healthy eating |
| 241 | LMC Diabetes & Endocrinology | 2022 | Sugars & Sweeteners | National NGO | Handout | None | No | Blood sugar management Weight management | Carbohydrate counting Label reading Non-nutritive sweeteners Macronutrients |
| 242 | LMC Diabetes & Endocrinology | 2017 | LMC Guide to Mindful Eating | National NGO | Handout | None | No | Blood sugar management Weight management Mental health | Meal planning |
| 243 | LMC Diabetes & Endocrinology | 2023 | LMC Guide to Gastroparesis | National NGO | Handout | None | No | Blood sugar management Weight management Gastroparesis Mental health | Macronutrients Meal planning Snack planning |
| 244 | LMC Diabetes & Endocrinology | 2021 | LMC Guide to Smart Snacking | National NGO | Handout | None | No | Blood sugar management Mental health | Glycemic index Macronutrients Snack planning |
| 245 | LMC Diabetes & Endocrinology | 2023 | Nutrition Tips to Get You Started | National NGO | Handout | None | No | Blood sugar management | Balanced plate method Macronutrients Meal planning Snack planning |
| 246 | LMC Diabetes & Endocrinology | nd | Meal Planning - South Asian | National NGO | Handout | None | South & Southeast Asian | Blood sugar management | Balanced plate method Macronutrients Meal planning Snack planning |
| 247 | National Aboriginal Diabetes Association | 2011 | Pathway to Wellness: A Handbook for Aboriginal People Living with Diabetes | National NGO | Handout | None | Indigenous | Blood sugar management Weight management Cardiovascular disease Gastrointestinal health Hypertension Neuropathy Retinopathy Mental health | Canada's Food Guide Balanced plate method Label reading Macronutrients Micronutrients Meal planning |
| 248 | National Indigenous Diabetes Association | 2020 | Gifts From Our Relations: Indigenous Original Foods Guide | National NGO | Report | French | Indigenous | Blood sugar management Cardiovascular disease Gastrointestinal health Hypertension Mental health | Macronutrients Micronutrients Meal planning |
| 249 | Native Women's Association of Canada | 2012 | Diabetes Self-Management Toolkit for Aboriginal Women | National NGO | Handout | None | Indigenous | Blood sugar management Weight management Cardiovascular disease Hypertension Mental health | Canada's Food Guide Balanced plate method Glycemic index Label reading Macronutrients Micronutrients Meal planning Snack planning Vegetarian diet |
| 250 | National Indigenous Diabetes Association | 2008 | Diabetes and Healthy Eating | P/T NGO | Handout | None | Indigenous | Blood sugar management | Balanced plate method Carbohydrate counting Non-nutritive sweeteners Macronutrients Micronutrients |
| 251 | Nova Scotia Health Authority | 2021 | Heart Healthy, Sodium Restricted Guidelines: 1500 to 2000 mg sodium per day | P/T gov. | Handout | None | No | Blood sugar management Cardiovascular disease Weight management Hypertension | Mediterranean diet Canada's Food Guide Balanced plate method Label reading Eating away from home Macronutrients Micronutrients Meal planning Snack planning |
| 252 | Nova Scotia Health Authority | 2022 | High Energy, High Protein Diabetic Guidelines | P/T gov. | Handout | None | No | Blood sugar management Weight management | Macronutrients Micronutrients Meal planning Snack planning |
| 253 | Nova Scotia Health Authority | 2020 | Gastroparesis Meal Planning | P/T gov. | Handout | None | No | Blood sugar management Gastrointestinal health Gastroparesis Nephropathy | Macronutrients Meal planning |
| 254 | Nova Scotia Health Authority | 2014 | Diabetes and Nutrition | P/T gov. | Web article | French | No | Blood sugar management | Macronutrients Meal planning Snack planning Label reading |
| 255 | Nuu-Chah-Nulth Tribal Council | 2020 | Sodium and Your Health | P/T NGO | Handout | None | Indigenous | Blood sugar management Cardiovascular disease Hypertension Nephropathy | Label reading Eating away from home Micronutrients Meal planning Snack planning |
| 256 | Nuu-Chah-Nulth Tribal Council | 2020 | Power Proteins | P/T NGO | Handout | None | Indigenous | Blood sugar management | Macronutrients Micronutrients General healthy eating |
| 257 | Nuu-Chah-Nulth Tribal Council | 2021 | Nuu-Chah-Nulth Seasonal Round | P/T NGO | Handout | None | Indigenous | Blood sugar management | General healthy eating |
| 258 | Nuu-Chah-Nulth Tribal Council | 2020 | Nutrition for High Blood Pressure | P/T NGO | Handout | None | Indigenous | Blood sugar management Cardiovascular disease Hypertension Nephropathy Retinopathy Mental health | DASH diet Label reading Eating away from home Macronutrients Micronutrients Meal planning Snack planning |
| 259 | Nuu-Chah-Nulth Tribal Council | 2022 | The Nuu-Chah-Nulth Food for Wellness Cookbook | P/T NGO | Handout | None | Indigenous | Blood sugar management Cardiovascular disease Gastrointestinal health Mental health | Macronutrients Micronutrients Meal planning Snack planning |
| 260 | Nuu-Chah-Nulth Tribal Council | 2019 | Healthy Granola Bars | P/T NGO | Handout | None | Indigenous | Blood sugar management | Label reading Macronutrients |
| 261 | Nuu-Chah-Nulth Tribal Council | nd | Food and Blood Sugars | P/T NGO | Handout | None | Indigenous | Blood sugar management | Macronutrients |
| 262 | Nuu-Chah-Nulth Tribal Council | 2019 | Carbohydrates and Diabetes | P/T NGO | Handout | None | Indigenous | Blood sugar management Cardiovascular disease | Glycemic index Macronutrients General healthy eating |
| 263 | Nuu-Chah-Nulth Tribal Council | nd | Healthy Snacks for Diabetes | P/T NGO | Handout | None | Indigenous | Blood sugar management Cardiovascular disease | Macronutrients Snack planning |
| 264 | Nuu-Chah-Nulth Tribal Council | 2019 | Taking Care of my Diabetes | P/T NGO | Handout | None | Indigenous | Blood sugar management Cardiovascular disease Hypertension Nephropathy Neuropathy Retinopathy Mental health | Macronutrients Micronutrients General healthy eating |
| 265 | Nuu-Chah-Nulth Tribal Council | 2020 | Nutrition for High Cholesterol | P/T NGO | Handout | None | Indigenous | Blood sugar management Cardiovascular disease Weight management | Label reading Macronutrients Meal planning Snack planning |
| 266 | Nuu-Chah-Nulth Tribal Council | 2019 | Healthy Eating on a Budget | P/T NGO | Handout | None | Indigenous | Blood sugar management Weight management | Vegetarian diet Macronutrients Micronutrients Meal planning |
| 267 | Regina Qu'Appelle Health Region | 2014 | Gastroparesis in Diabetes (Slow Stomach Emptying) | P/T NGO | Handout | None | No | Blood sugar management Gastroparesis Neuropathy | Macronutrients Meal planning |
| 268 | Saskatchewan Health Authority | 2020 | Carbohydrate Content of Foods | P/T gov. | Handout | None | No | Blood sugar management Cardiovascular disease | Canada's Food Guide Carbohydrate counting Label reading Eating away from home Non-nutritive sweeteners Macronutrients Micronutrients Meal planning |
| 269 | Saskatchewan Health Authority, Healthwise | 2022 | Diabetes - How to build your plate | P/T gov. | Audiovisual | None | No | Blood sugar management | Balanced plate method Macronutrients |
| 270 | The Community Diabetes Education Program of Ottawa | 2018 | The Diabetes Food Guide to Healthy Eating | P/T NGO | Handout | Arabic; French; Italian; Chinese (Simplified); Spanish; Vietnamese | No | Blood sugar management | Macronutrients General healthy eating Meal planning |
| 271 | University Health Network (Diabetes College) | 2018 | Choosing Healthy Foods: Choosing a Healthy Cereal | P/T gov. | Handout | None | No | Blood sugar management | Label reading Macronutrients Micronutrients General healthy eating |
| 272 | University Health Network (Diabetes College) | 2018 | Choosing Healthy Foods: Choosing Seafood and Fish | P/T gov. | Handout | None | No | Blood sugar management | Label reading Macronutrients Micronutrients General healthy eating |
| 273 | University Health Network (Diabetes College) | 2018 | Choosing Healthy Foods: Choosing Healthy Snack Foods | P/T gov. | Handout | None | No | Blood sugar management | Label reading Macronutrients Micronutrients  General healthy eating |
| 274 | University Health Network (Diabetes College) | 2018 | Choosing Healthy Foods: Choosing a Healthy Soup | P/T gov. | Handout | None | No | Blood sugar management | Label reading Macronutrients Micronutrients General healthy eating |
| 275 | University Health Network (Diabetes College) | 2018 | Choosing Healthy Foods: Choosing Soy Protein | P/T gov. | Handout | None | No | Blood sugar management | Macronutrients Micronutrients General healthy eating |
| 276 | University Health Network (Diabetes College) | 2018 | Choosing Healthy Foods: Choosing Healthy Spices | P/T gov. | Handout | None | No | Blood sugar management Cardiovascular disease | Label reading Micronutrients General healthy eating |
| 277 | University Health Network (Diabetes College) | 2018 | Choosing Healthy Foods: Choosing Vegetables and Fruits | P/T gov. | Handout | None | No | Blood sugar management | Label reading Micronutrients General healthy eating |
| 278 | University Health Network (Diabetes College) | 2018 | Choosing Healthy Foods: Choosing a Healthy Yogurt | P/T gov. | Handout | None | No | Blood sugar management | Label reading Macronutrients General healthy eating |
| 279 | University Health Network (Diabetes College) | 2018 | Choosing Healthy Foods: Choosing Healthy Meats | P/T gov. | Handout | None | No | Blood sugar management | Macronutrients Micronutrients General healthy eating |
| 280 | University Health Network (Diabetes College) | 2018 | Choosing Healthy Foods: Choosing Milk and Non-Dairy Alternatives | P/T gov. | Handout | None | No | Blood sugar management | Label reading Macronutrients General healthy eating |
| 281 | University Health Network (Diabetes College) | 2018 | Choosing Healthy Foods: Choosing Nuts and Nut Butters | P/T gov. | Handout | None | No | Blood sugar management | Label reading Macronutrients General healthy eating |
| 282 | University Health Network (Diabetes College) | 2018 | Choosing Healthy Foods: Choosing a Healthy Pasta | P/T gov. | Handout | None | No | Blood sugar management | Label reading Macronutrients Micronutrients General healthy eating |
| 283 | University Health Network (Diabetes College) | 2018 | Choosing Healthy Foods: Choosing Healthy Sauces, Condiments, and Salad Dressings | P/T gov. | Handout | None | No | Blood sugar management | Label reading Macronutrients Micronutrients General healthy eating |
| 284 | University Health Network (Diabetes College) | 2018 | Choosing Healthy Foods: Choosing Legumes | P/T gov. | Handout | None | No | Blood sugar management | Label reading Macronutrients Micronutrients General healthy eating |
| 285 | University Health Network (Diabetes College) | 2018 | Choosing Healthy Foods: Choosing Healthy Frozen Foods | P/T gov. | Handout | None | No | Blood sugar management | Label reading Macronutrients Micronutrients General healthy eating |
| 286 | University Health Network (Diabetes College) | 2018 | Choosing Healthy Foods: Choosing Healthy Drinks | P/T gov. | Handout | None | No | Blood sugar management | Label reading Non-nutritive sweeteners General healthy eating |
| 287 | University Health Network (Diabetes College) | 2018 | Choosing Healthy Foods: Choosing a Healthy Cooking Oil | P/T gov. | Handout | None | No | Blood sugar management Cardiovascular disease | Macronutrients General healthy eating |
| 288 | University Health Network (Diabetes College) | 2018 | Choosing Healthy Foods: Choosing a Healthy Bread | P/T gov. | Handout | None | No | Blood sugar management | Label reading Macronutrients Micronutrients General healthy eating |
| 289 | University Health Network (Diabetes College) | 2018 | Choosing Healthy Foods: Choosing a Healthier Cheese | P/T gov. | Handout | None | No | Blood sugar management | Label reading Macronutrients Micronutrients General healthy eating |
| 290 | University Health Network (Diabetes College) | 2020 | How to Eat to Help Manage Your Diabetes - Getting Started | P/T gov. | Handout | None | No | Blood sugar management | Balanced plate method Macronutrients |
| 291 | University Health Network (Diabetes College) | 2018 | Diabetes Plate Model | P/T gov. | Web article | French | No | Blood sugar management | Balanced plate method Glycemic index Macronutrients Meal planning |
| 292 | Vancouver Coastal Health | 2012 | Eating Healthy for People with Diabetes and Kidney Disease (Non-Dialysis) | P/T gov. | Handout | None | No | Blood sugar management Nephropathy | Balanced plate method Non-nutritive sweeteners Macronutrients Micronutrients Meal planning Snack planning |
| 293 | Island Health | 2013 | Setting the Table: Enjoying, Exploring & Sharing Food | P/T gov. | Report | None | Indigenous | Blood sugar management Cardiovascular disease Gastrointestinal health Hypertension | Macronutrients Micronutrients Meal planning Snack planning |
| 294 | Island Health | 2018 | Balance Your Plate - Lunch and Dinner | P/T gov. | Handout | None | No | Blood sugar management | Balanced plate method Macronutrients |
| 295 | Island Health, W̱SÁNEĆ Leadership Secondary | 2018 | Food is Medicine: Cooking Together 2016-2018 | P/T NGO | Handout | None | Indigenous | Blood sugar management Cardiovascular disease | Macronutrients Micronutrients Meal planning Snack planning |

# Geographic location of knowledge experts (RD CDEs) consulted

| Province or Territory | City or Town | Size of Population Centre^*^ | Number of Knowledge Experts | |
| --- | --- | --- | --- | --- |
|  |  |  | By City or Town | By Province or Territory |
| British Columbia | Vancouver | Large | 2 | 4 |
|  | Victoria | Large | 1 |  |
|  | Parksville | Small | 1 |  |
| Alberta | Calgary | Large | 2 | 3 |
|  | Canmore | Small | 1 |  |
| Saskatchewan | Saskatoon | Large | 1 | 2 |
|  | Lloydminster | Medium | 1 |  |
| Manitoba | Winnipeg | Large | 1 | 1 |
| Ontario | Toronto | Large | 4 | 4 |
| Quebec | Montreal | Large | 4 | 4 |
| Newfoundland and Labrador | St. John’s | Large | 1 | 1 |
| New Brunswick | Moncton | Medium | 1 | 3 |
|  | Bathurst | Small | 1 |  |
|  | Campbellton | Small | 1 |  |
| Nova Scotia | Halifax | Large | 1 | 1 |
| Prince Edward Island | Charlottetown | Medium | 2 | 2 |
| Nunavut | Iqaluit | Small | 1 | 1 |
| Total Number of Knowledge Experts | | | | 26 |

# Detailed comparison of descriptive characteristics among resources by cultural specificity and language availability

|  | Professional Resources | | | | | Patient Resources | | | | | | | | | | | | | | |
| --- | --- | --- | --- | --- | --- | --- | --- | --- | --- | --- | --- | --- | --- | --- | --- | --- | --- | --- | --- | --- |
|  | Culturally Specific | | | | | Culturally Specific | | | | | Third Language  Translations Available | | | | | Culturally Specific and Third Language Translations Available | | | | |
|  | Yes | | No | | P-value | Yes | | No | | P-value | Yes | | No | | P-value | Yes | | No | | P-value |
| n (%) of all resources | 17 | (37) | 29 | (63) | 0.204 | 84 | (28) | 211 | (72) | <0.001^***^ | 28 | (9) | 267 | (91) | <0.001^***^ | 11 | (4) | 284 | (96) | <0.001^***^ |
| Year Published |  |  |  |  |  |  |  |  |  |  |  |  |  |  |  |  |  |  |  |  |
| 2018 to 2023 | 12 | (71) | 27 | (93) | 0.083 | 40 | (48) | 151 | (72) | <0.001^***^ | 20 | (71) | 171 | (64) | 0.535 | 7 | (64) | 184 | (65) | 1.000 |
| 2012 to 2017 | 4 | (24) | 1 | (3) | 0.055 | 32 | (38) | 42 | (20) | 0.002^**^ | 5 | (18) | 69 | (26) | 0.492 | 1 | (9) | 73 | (35) | 0.302 |
| 2007 to 2011 | 1 | (6) | 0 | (0) | 0.37 | 7 | (8) | 5 | (2) | 0.043^*^ | 3 | (11) | 9 | (3) | 0.094 | 3 | (27) | 9 | (4) | 0.007^**^ |
| Unknown | 0 | (0) | 1 | (3) | 1.000 | 5 | (6) | 7 | (3) | 1.000 | 0 | (0) | 12 | (4) | 0.613 | 0 | (0) | 12 | (6) | 1.000 |
| Type of Organization |  |  |  |  |  |  |  |  |  |  |  |  |  |  |  |  |  |  |  |  |
| National NGO | 13 | (76) | 23 | (79) | 1.000 | 44 | (52) | 89 | (42) | 0.121 | 19 | (68) | 114 | (43) | 0.016^*^ | 8 | (73) | 125 | (59) | 0.071 |
| National gov. | 0 | (0) | 0 | (0) | 1.000 | 2 | (2) | 1 | (0) | 0.190 | 1 | (4) | 2 | (1) | 0.259 | 1 | (9) | 2 | (1) | 0.108 |
| P/T NGO | 0 | (0) | 1 | (3) | 1.000 | 24 | (29) | 79 | (37) | 0.176 | 4 | (14) | 99 | (37) | 0.020^*^ | 0 | (0) | 103 | (49) | 0.010^**^ |
| P/T gov. | 4 | (24) | 5 | (17) | 0.707 | 14 | (17) | 42 | (20) | 0.622 | 0 | (0) | 56 | (21) | 0.004^**^ | 0 | (0) | 56 | (27) | 0.133 |
| Type of Resource |  |  |  |  |  |  |  |  |  |  |  |  |  |  |  |  |  |  |  |  |
| Clinical practice guideline | 6 | (35) | 18 | (62) | 0.126 | 0 | (0) | 0 | (0) | 1.000 | 0 | (0) | 0 | (0) | 1.000 | 0 | (0) | 0 | (0) | 1.000 |
| Position Statement | 1 | (6) | 1 | (3) | 1.000 | 0 | (0) | 0 | (0) | 1.000 | 0 | (0) | 0 | (0) | 1.000 | 0 | (0) | 0 | (0) | 1.000 |
| Report | 0 | (0) | 5 | (17) | 0.142 | 5 | (6) | 1 | (0) | 0.008^**^ | 0 | (0) | 6 | (2) | 1.000 | 0 | (0) | 6 | (3) | 1.000 |
| Handout | 6 | (35) | 1 | (3) | 0.007** | 70 | (83) | 132 | (63) | <0.001^***^ | 28 | (100) | 174 | (65) | <0.001^***^ | 11 | (100) | 191 | (91) | 0.019^*^ |
| Web article | 0 | (0) | 0 | (0) | 1.000 | 4 | (5) | 67 | (32) | <0.001^***^ | 0 | (0) | 71 | (27) | <0.001^***^ | 0 | (0) | 71 | (34) | 0.072 |
| Audiovisual | 4 | (24) | 4 | (14) | 0.443 | 5 | (6) | 11 | (5) | 0.781 | 0 | (0) | 16 | (6) | 0.379 | 0 | (0) | 16 | (8) | 1.000 |
| Disease management goals |  |  |  |  |  |  |  |  |  |  |  |  |  |  |  |  |  |  |  |  |
| Blood sugar management | 17 | (100) | 29 | (100) | 1.000 | 84 | (100) | 211 | (100) | 1.000 | 28 | (100) | 267 | (100) | 1.000 | 11 | (100) | 284 | (100) | 1.000 |
| Weight management | 9 | (53) | 26 | (90) | 0.010* | 24 | (29) | 65 | (31) | 0.779 | 9 | (32) | 80 | (30) | 0.826 | 2 | (18) | 87 | (41) | 0.514 |
| Cardiovascular disease | 8 | (47) | 25 | (86) | 0.007** | 34 | (40) | 77 | (36) | 0.594 | 12 | (43) | 99 | (37) | 0.546 | 2 | (18) | 109 | (52) | 0.218 |
| Hypertension | 6 | (35) | 19 | (66) | 0.068 | 18 | (21) | 33 | (16) | 0.237 | 5 | (18) | 46 | (17) | 1.000 | 0 | (0) | 51 | (24) | 0.222 |
| Gastrointestinal health | 0 | (0) | 1 | (3) | 1.000 | 11 | (13) | 27 | (13) | 1.000 | 1 | (4) | 37 | (14) | 0.148 | 0 | (0) | 38 | (18) | 0.370 |
| Gastroparesis | 0 | (0) | 1 | (3) | 1.000 | 0 | (0) | 6 | (3) | 0.188 | 0 | (0) | 6 | (2) | 1.000 | 0 | (0) | 6 | (3) | 1.000 |
| Nephropathy | 5 | (29) | 11 | (38) | 0.750 | 11 | (13) | 16 | (8) | 0.178 | 0 | (0) | 27 | (10) | 0.090 | 0 | (0) | 27 | (13) | 0.607 |
| Neuropathy | 1 | (6) | 2 | (7) | 1.000 | 6 | (7) | 9 | (4) | 0.378 | 2 | (7) | 13 | (5) | 0.642 | 0 | (0) | 15 | (7) | 1.000 |
| Retinopathy | 1 | (6) | 2 | (7) | 1.000 | 7 | (8) | 8 | (4) | 0.140 | 2 | (7) | 13 | (5) | 0.642 | 0 | (0) | 15 | (7) | 1.000 |
| Mental health | 9 | (53) | 17 | (59) | 0.765 | 21 | (25) | 17 | (8) | <0.001^***^ | 6 | (21) | 32 | (12) | 0.229 | 3 | (27) | 35 | (17) | 0.400 |
| MNT Topics |  |  |  |  |  |  |  |  |  |  |  |  |  |  |  |  |  |  |  |  |
| General healthy eating | 5 | (29) | 2 | (7) | 0.083 | 10 | (12) | 51 | (24) | 0.025^*^ | 4 | (14) | 57 | (21) | 0.469 | 1 | (9) | 60 | (28) | 0.470 |
| Meal planning | 10 | (59) | 6 | (21) | 0.012* | 56 | (67) | 100 | (47) | 0.003^**^ | 17 | (61) | 139 | (52) | 0.43 | 7 | (64) | 149 | (71) | 0.549 |
| Snack planning | 5 | (29) | 4 | (14) | 0.258 | 51 | (61) | 82 | (39) | <0.001^***^ | 15 | (54) | 118 | (44) | 0.376 | 7 | (64) | 126 | (60) | 0.232 |
| Macronutrients | 11 | (65) | 25 | (86) | 0.139 | 74 | (88) | 188 | (89) | 0.839 | 24 | (86) | 238 | (89) | 0.534 | 10 | (91) | 252 | (119) | 1.000 |
| Micronutrients | 6 | (35) | 17 | (59) | 0.221 | 34 | (40) | 78 | (37) | 0.597 | 7 | (25) | 105 | (39) | 0.154 | 4 | (36) | 108 | (51) | 1.000 |
| Carbohydrate counting | 1 | (6) | 4 | (14) | 0.637 | 11 | (13) | 46 | (22) | 0.103 | 10 | (36) | 47 | (18) | 0.040^*^ | 5 | (45) | 52 | (25) | 0.041^*^ |
| Label reading | 2 | (12) | 6 | (21) | 0.691 | 29 | (35) | 91 | (43) | 0.191 | 8 | (29) | 112 | (42) | 0.225 | 0 | (0) | 120 | (57) | 0.004^**^ |
| Eating away from home | 0 | (0) | 2 | (7) | 0.524 | 19 | (23) | 28 | (13) | 0.054 | 8 | (29) | 39 | (15) | 0.098 | 4 | (36) | 43 | (20) | 0.080 |
| Non-nutritive sweeteners | 2 | (12) | 6 | (21) | 0.691 | 8 | (10) | 36 | (17) | 0.107 | 6 | (21) | 38 | (14) | 0.278 | 3 | (27) | 41 | (19) | 0.216 |
| Dietary patterns | 9 | (53) | 20 | (69) | 0.349 | 54 | (64) | 93 | (44) | 0.002^**^ | 21 | (75) | 126 | (47) | 0.005^**^ | 10 | (91) | 137 | (65) | 0.005^**^ |
| Mediterranean diet | 2 | (12) | 12 | (41) | 0.049* | 3 | (4) | 10 | (5) | 0.764 | 2 | (7) | 11 | (4) | 0.355 | 1 | (9) | 12 | (6) | 0.396 |
| Glycemic index | 6 | (35) | 10 | (34) | 1.000 | 15 | (18) | 31 | (15) | 0.483 | 8 | (29) | 38 | (14) | 0.057 | 3 | (27) | 43 | (20) | 0.387 |
| Low carbohydrate diet | 2 | (12) | 15 | (52) | 0.011* | 0 | (0) | 2 | (1) | 1.000 | 0 | (0) | 2 | (1) | 1.000 | 0 | (0) | 2 | (1) | 1.000 |
| Canada's Food Guide | 3 | (18) | 7 | (24) | 0.727 | 23 | (27) | 26 | (12) | 0.003^**^ | 7 | (25) | 42 | (16) | 0.282 | 4 | (36) | 45 | (21) | 0.091 |
| Intermittent fasting | 1 | (6) | 6 | (21) | 0.234 | 0 | (0) | 1 | (0) | 1.000 | 0 | (0) | 1 | (0) | 1.000 | 0 | (0) | 1 | (0) | 1.000 |
| Low calorie diet | 0 | (0) | 8 | (28) | 0.019* | 0 | (0) | 0 | (0) | 1.000 | 0 | (0) | 0 | (0) | 1.000 | 0 | (0) | 0 | (0) | 1.000 |
| DASH diet | 1 | (6) | 9 | (31) | 0.067 | 5 | (6) | 8 | (4) | 0.564 | 3 | (11) | 10 | (4) | 0.115 | 1 | (9) | 12 | (6) | 0.396 |
| Low fat diet | 1 | (6) | 9 | (31) | 0.067 | 0 | (0) | 0 | (0) | 1.000 | 0 | (0) | 0 | (0) | 1.000 | 0 | (0) | 0 | (0) | 1.000 |
| Nordic diet | 1 | (6) | 1 | (3) | 1.000 | 2 | (2) | 0 | (0) | 0.214 | 1 | (4) | 1 | (0) | 0.259 | 1 | (9) | 1 | (0) | 0.073 |
| Popular weight loss diets | 1 | (6) | 4 | (14) | 0.637 | 0 | (0) | 2 | (1) | 1.000 | 2 | (7) | 0 | (0) | <0.001^***^ | 0 | (0) | 2 | (6) | 1.000 |
| Balanced Plate Method | 3 | (18) | 2 | (7) | 0.637 | 31 | (37) | 48 | (23) | 0.019^*^ | 13 | (46) | 66 | (25) | 0.023^**^ | 5 | (45) | 74 | (35) | 0.171 |
| Gluten-free diet | 0 | (0) | 0 | (0) | 0.359 | 2 | (2) | 4 | (2) | 1.000 | 0 | (0) | 6 | (2) | 1.000 | 0 | (0) | 6 | (3) | 1.000 |
| Vegetarian diet | 1 | (6) | 7 | (24) | 0.226 | 12 | (14) | 11 | (5) | 0.014^*^ | 2 | (0) | 21 | (8) | 1.000 | 2 | (18) | 21 | (10) | 0.208 |
| Portfolio diet | 1 | (6) | 4 | (14) | 0.637 | 1 | (1) | 2 | (1) | 1.000 | 0 | (0) | 3 | (1) | 1.000 | 0 | (0) | 3 | (1) | 1.000 |

P/T, provincial or territorial. Gov., government*.* Data expressed as number (percent of total resources per column) unless otherwise stated. Comparisons made using Fisher’s exact test between proportions of resources in the “Yes” and “No” columns of each resource category. ^*^ p < 0.05 ^**^ p < 0.01 *** p < 0.001

#
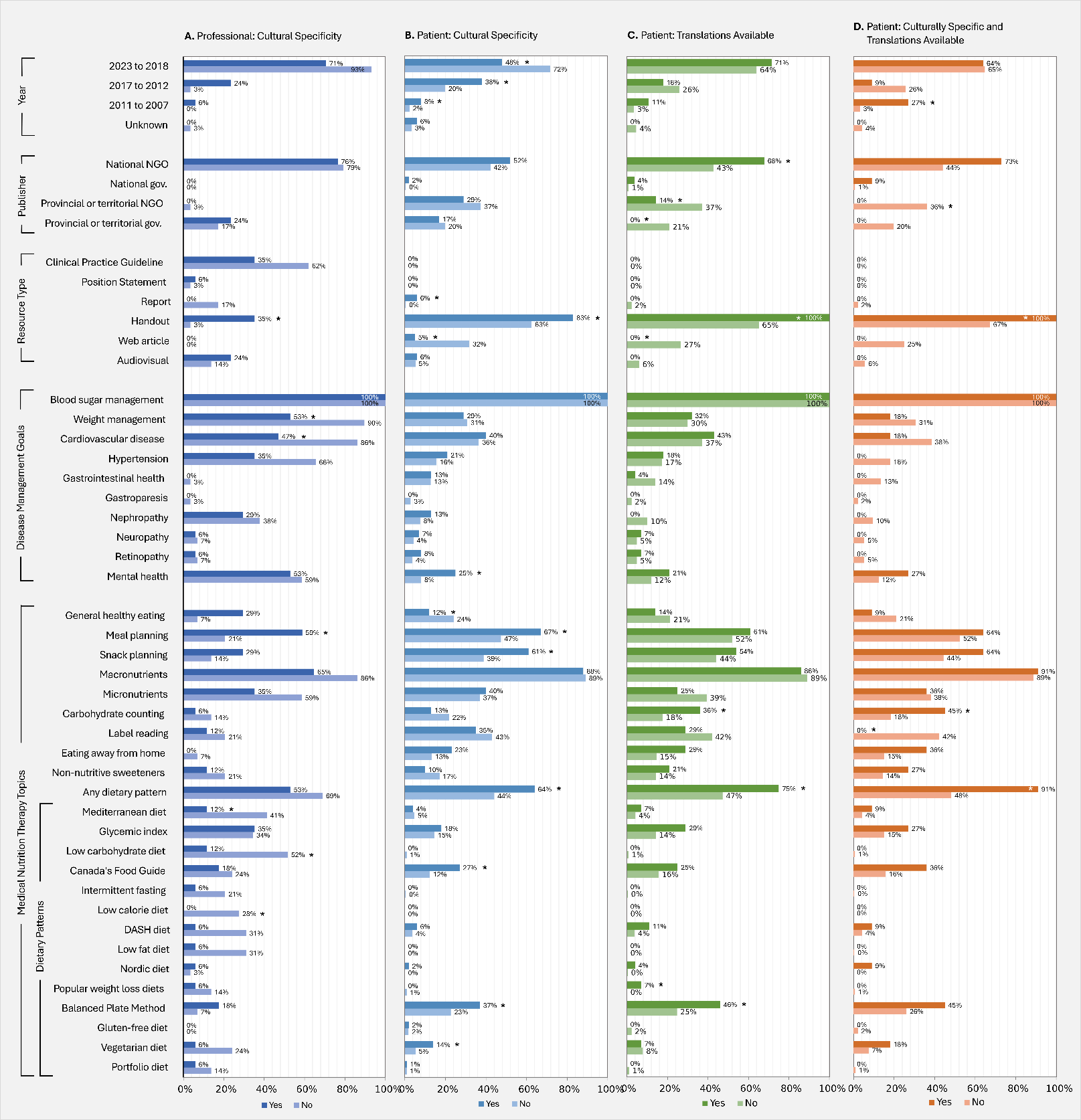
Figure comparing descriptive characteristics among resources by cultural specificity and language availability

Comparison of descriptive characteristics and inclusion of nutrition management of diabetes-related comorbidities and medical nutrition therapy topics among (A) professional resources with and without cultural specificity (n=17 versus n=29, respectively), (B) patient resources with and without specificity (n=84 versus n=211), (C) patient resources with and without third-language translations (n=28 versus n=267), and (D) patient resources with and without both cultural specificity and third-language translations (n=11 versus n=284). Comparisons between professional (patient) resources with and without cultural specificity (language availability) were conducted using Fisher’s Exact test. * Indicates statistically significant differences at p<0.05.

1. At the time of data collection, most of Vancouver Coastal Health handouts were under revision and not available for public access online. [↑](#footnote-ref-1)
